# Cosolvent Analysis Toolkit (CAT): a robust hotspot identification platform for cosolvent simulations of proteins to expand the druggable proteome

**DOI:** 10.1038/s41598-019-55394-2

**Published:** 2019-12-13

**Authors:** Francesc Sabanés Zariquiey, João V. de Souza, Agnieszka K. Bronowska

**Affiliations:** Chemistry – School of Natural and Environmental Sciences, Newcastle University, NE1 7RU Newcastle, United Kingdom

**Keywords:** Computational models, Computational platforms and environments, Protein function predictions, Software, Molecular modelling

## Abstract

Cosolvent Molecular Dynamics (MD) simulations are increasingly popular techniques developed for prediction and characterization of allosteric and cryptic binding sites, which can be rendered “druggable” by small molecule ligands. Despite their conceptual simplicity and effectiveness, the analysis of cosolvent MD trajectories relies on pocket volume data, which requires a high level of manual investigation and may introduce a bias. In this work, we present CAT (Cosolvent Analysis Toolkit): an open-source, freely accessible analytical tool, suitable for automated analysis of cosolvent MD trajectories. CAT is compatible with commonly used molecular graphics software packages such as UCSF Chimera and VMD. Using a novel hybrid empirical force field scoring function, CAT accurately ranks the dynamic interactions between the macromolecular target and cosolvent molecules. To benchmark, CAT was used for three validated protein targets with allosteric and orthosteric binding sites, using five chemically distinct cosolvent molecules. For all systems, CAT has accurately identified all known sites. CAT can thus assist in computational studies aiming at identification of protein “hotspots” in a wide range of systems. As an easy-to-use computational tool, we expect that CAT will contribute to an increase in the size of the potentially ‘druggable’ human proteome.

## Introduction

Over 75% of proteins relevant as disease targets cannot be readily targeted by conventional structure-based and chemical biology approaches^1,2^. Cryptic binding pockets, i.e. pockets that form in a protein upon ligand binding, but are not apparent in the crystal structure of the *apo* (unliganded) protein, and transient pockets, i.e. pockets with transiently form in a subset of an ensemble of protein conformations, offer immense opportunities to target proteins deemed ‘undruggable’ by conventional structure-based drug design (SBDD) approaches and are thus of considerable interest in academia and the pharmaceutical industry. Unfortunately, these ‘hotspots’ are not only notoriously difficult to identify, but the molecular mechanisms by which they form are still debated^2,3^.

Protein ‘hotspots’ are usually comprised of a set of residues that make a considerable contribution to the binding free energy. In past years, identifying them has been considered a key to target ‘undruggable’ proteins. Techniques such as Multiple Solvent Crystal Structures (MSCS)^4^, alanine scanning^5^ and structure-activity relationship by nuclear magnetic resonance (SAR by NMR)^6^ have been able to identify hotspots in a number of proteins. These methods tend to be highly resource- and time consuming, therefore the interest in developing computational tools able to identify ‘hotspots’ has emerged, resulting in approaches involving machine learning^6^ and Multiple Copy Simultaneous Search (MCSS)^7^, among others. Methods involving machine learning rely on experimental data, i.e. cryptic pockets solved by X-ray crystallography, whose number is very limited^8^. The major shortcoming of MCSS is the fact that the probes do not interact with one another, which results in the loss of any possible cooperativity in their binding. Another limitation lies in the static structure of the protein target analyzed: any ligand-induced conformational changes cannot be observed, which precludes its applicability to the identification of cryptic and transient pockets.

An approach devoid of these shortcomings is all-atom molecular dynamics (MD) simulation. However, attempts of identifying ‘hotspots’ by conventional MD simulations in an aqueous solvent are limited by relatively short timescales accessible (nanoseconds to single-digit microseconds), precluding observation of significant conformational changes that occur on microseconds to milliseconds timescales.

 Enhanced sampling techniques have proven effective at overcoming the timescale limitations of conventional equilibrium MD simulations and have successfully sampled cryptic pocket formation in several recently reported cases^9^, but those methods are restricted to the community of specialists in molecular simulations. To overcome the accessibility problem, easy to use tools for non-experts offering scans for potential cryptic, allosteric, and transient pockets have been established and they have gained popularity in recent years^10–14^.

One of the most common pocket detection tools is the FTMap^15^ webserver. A fast, easy to use method based on the sampling of a series of probe molecules ranked by an interaction-druggability scoring function, resulting in a set of top clusters. Though FTMap achieves a remarkable agreement with experimental data^15,16^, it presents some caveats. Mainly, the lack of a longer sampling through dynamics affecting the overall cleft formation, which restricts its ability to identify new cryptic binding sites.

An alternative yet simple approach to map molecular hotspots is relying on cosolvent MD simulations. This technique, involving simulating the target protein in a mixture of small molecular fragments (cosolvents) and water, was introduced in 2009^17^, and it is being increasingly applied towards the discovery of novel binding sites and structure-based development of small molecule allosteric inhibitors. Success stories of cosolvent MD simulations include MixMD^18,19^, MDMix^20^, SILCS^21^, and others^22^. Simulations containing multiple fragment types require fewer simulations than comparable methods that simulate each fragment separately, but the extent to which this influences the predicted binding sites remains unclear.

Cosolvent MD simulations, while being straightforward to carry out by experts and non-experts alike, suffer similar shortcomings as the conventional MD simulation: namely, these are limited by the timescale. In addition, hydrophobic fragments tend to aggregate during cosolvent MD runs, which imposes constraints on the concentrations used^23^.

Another factor which hampers a wide use of cosolvent MD approaches is within data analysis. Traditionally, analysis of cosolvent MD simulations required a great deal of manual inspection to identify relevant sites, which made those simulations difficult to use for medicinal chemists and structural biologists. To enable fast and efficient cosolvent MD simulation data analysis, we have developed CAT (Cosolvent Analysis Toolkit). CAT has been designed as an open-source analytical platform, compatible with commonly used molecular graphics software packages such as UCSF Chimera^24^ and VMD^25^. This feature is similar to Graham’s Probeview^19^, in which a PyMol plugin is developed to analyze the results of their cosolvent analysis approach. Conventionally, protein pockets have been identified by overlapping density from multiple fragment molecules. Such approach, although straightforward to implement, is prone to certain errors arising from e.g. stacking of aromatic of hydrophobic fragments due to hydrophobic effect.

CAT relies on a robust hybrid empirical-force field scoring function, which uses a softcore potential^26^ to the non-covalent interaction energy terms (see Methods). By utilising it, CAT avoids several pitfalls commonly found within the analysis of cosolvent MD trajectories. First, the softcore potential smooths the interaction energy landscape, fending off transient atomic clashes and improving the overall receptor-ligand shape complementarity. Along with that, the energy score implemented in CAT includes the regional average depth and the cosolvent retention time in the respective region. This results in an accurate measurement of the dynamical effects of binding directly incorporated in the ranking by CAT.

CAT incorporates two types of analysis: identification and ranking of the entire ‘hotspots’, and identification and ranking of the molecular fragments suitable for targeting those ‘hotspots’. The former serves as a general detector and can be readily used to guide structural biology experimental efforts, while the latter brings useful information about the inhibitor/ligand design from the structure-guided standpoint. The performance of CAT has been validated using four benchmark proteins: H-Ras GTPase, protein tyrosine phosphatase 1B (PTP1B), ligand binding domain of human androgen receptor (AR-LBD) and cyclin-dependent kinase 2 (CDK2). Two of these proteins have both orthosteric and allosteric binding sites. The results obtained were compared to the results obtained by FTMap. For all systems tested, CAT successfully identified allosteric sites, which were challenging to FTMap.

## Methods

### Selection of the benchmarking molecules and probes

To assess CAT’s efficiency on filtering and ranking hotspots, a set of probe molecules and protein targets were selected. Probe molecules (acetamide, benzene, acetanilide, imidazole and isopropanol) were selected based on three criteria: first, this set has a range of solubility characteristics, going from fully hydrophobic molecules such as benzene to more hydrophilic molecules like acetamide. Second, all the probes are widely used molecular fragments, as crystallization co-factors, probes employed in fragment-based drug discovery (FBDD) efforts, and as moieties present in known small molecule ligands. Third, it is a set validated in previously reported studies on allosteric hotspot mapping^16,19,22,27^.

Regarding the target selection, our focus was set on proteins with reported crystallographic structures of their orthosteric binding site with more than one reported allosteric site; which have been used in benchmarks of similar techniques^28^. After a careful curation, four targets were selected: the ligand-binding domain of the androgen receptor (AR-LBD), protein-tyrosine phosphatase 1B (PTP1B) and GTPase HRas. Additionally, the cyclin-dependent kinase 2 (CDK2) has also been tested, as novel allosteric sites have been recently described^29^. As this set includes members of four distinct protein families, there is no bias towards any protein family in this benchmark.

### Structure preparation

The crystal structures used as starting conformations for the cosolvent MD simulations were in the *apo* state, whenever available (PDB codes are listed in Table 1). Structures were stripped of water molecules and any present cofactors and/or ligands. For structures with missing loops, the MODELLER^30^ interface in UCSF Chimera^24^ was used to rebuild the missing fragments. The best ZDOPE scored loops were selected to complete the model.

**Table 1:** PDB codes of the crystal structures used for our benchmarking, codes highlighted in bold correspond to the structures used for the cosolvent simulations.

Molecule	Starting structure	Benchmark structures
AR Ligand Binding Domain (AR-LBD)	**2PIO**	2PIQ, 2PIR, 2PIT, 2PIU, 2PIV, 2PIW, 2PIX, 2PKL
Protein-tyrosine phosphatase 1B (PTP1B)	**1XBO**	1T4J^42^, 1T48, 6B95^43^
GTPase HRas (HRas)	**1P2S**	1P2T, 1P2U, 1P2V, 3K8Y, 3K9L, 3K9N, 3RRZ, 3RS0, 3RS2, 3RS3, 3RS4, 3RS5, 3RS7
Cyclin-dependent Kinase 2 (CDK2)	**4EK3**	6Q3C, 6Q3B, 6Q3F, 6Q49, 6Q48,6Q4B, 6Q4A, 6Q4C, 6Q4D, 6Q4F, 6Q4E, 6Q4J, 6Q4I, 6Q4H, 6Q4G, 6Q4K.

Incomplete side chains were replaced using the Dunbrack rotamer library^31^, implemented in UCSF Chimera. For side chains with multiple locations, the highest occupancy conformations have been selected. Structural hydrogens were added and the following protein parametrization was performed using the Gromacs 2016.03^32^ suite with AMBERFF99SB-ILDN^33^ force field. A cubic box was centered around the protein target with 1 nm distance between the protein extreme to the edge. A pre-defined number of molecular probes (cosolvent molecules) were randomly inserted into the system, ensuring that after the following solvation with TIP3P waters there was a 10% (m/m) probe concentration in water in order to avoid phase separation and/or probe clustering. Each simulation used a single type of cosolvent molecule. The probe selection criteria consisted of using a series of drug-like small molecular fragments with a broad range of relevant properties, including hydrophilicity/hydrophobicity, aromaticity, and the number of hydrogen-bonding acceptors/donors, with a diverse range of logP values. The following molecules were used: acetamide, benzene, acetanilide, imidazole and isopropanol. To diminish the effect of phase separation and π−π stacking of aromatic and highly hydrophobic cosolvent molecules such as benzene, an approach similar to Mackerell and colleagues was chosen, which relied on placing a dummy atom with a negligible negative charge (e = −0.01) in the center of the 6-membered ring. All probes were parametrized using GAFF^34^ with AM1-BCC^35^ charges assigned by ACPYPE/ANTECHAMBER^36^.

### MD simulation protocol

Sodium and chloride ions were added to a concentration of 0.1 M. Bonds were constrained using the LINCS^37^ algorithm, with a 2 fs time step. The electrostatic interactions were calculated using the particle-mesh Ewald method, with a non-bonded cut-off set at 0.1 nm. All structures were minimized via the steepest descent algorithm for 20000 steps was stopped when the maximum force fell below 1000 kJ/mol/nm using the Verlet cutoff scheme. After the minimization, heating via NVT ensemble was performed for 100 ps with a time step of 2 fs with position restraints (1000 kJ/mol/nm^2^, applied in all three dimensions) applied to the backbone. The temperature coupling was set between the protein and the non-protein entities by using a Berendsen thermostat, with a time constant of 0.1 ps and the temperature set to reach 300 K with the pressure coupling off. Sequentially, a pressure NPT ensemble equilibration was performed followed by 100 ps, and three NPT ensemble production run replicas of 50 ns, totalling 150 ns for each different combination of protein and cosolvents, including the control simulations that are comprised of only protein-water systems. All production runs were unrestrained simulations^38^. The temperature was set constant at 300 K by using a modified Berendsen thermostat(τ = 0.1 ps)^39^. Pressure was kept constant at 1 bar by Parinello-Rahman isotropic coupling (τ = 2.0 ps) to a pressure bath.

Data analysis has initially been done within the Gromacs package. For each data set, the analysis involved calculating root-mean-square deviation (RMSD), root-mean-square fluctuations (RMSF), the covariance matrices and principal component analysis (PCA) and solvent accessible surface area (SASA) to analyze convergence of the runs. Afterwards, CAT was employed for every dataset to identify any potentially ‘druggable’ hotspots. To assess the convergence, the cluster centroids (Supp. Figs. 13–16) and radial distribution functions (RDF; Supp. Figs. 17–20) were calculated for each replica, for all cosolvent simulations.

### CAT analysis and the description of the scoring function

To create a reliable analytical method for the detection of molecular hotspots, the development of a scoring function was required. From a molecular interaction standpoint, such scoring function should include three characteristics: calculation of the interaction energy between the protein and cosolvent molecules, the retention time of the cosolvent molecule at the binding site, and the overall depth of the binding site relative to the protein surface. Therefore, the scoring function per residue can be written as follows (Eq. 1):

1 $${S}_{Residue}={S}_{Interaction}{S}_{Stability}{S}_{Depth}$$

**Figure 1:**
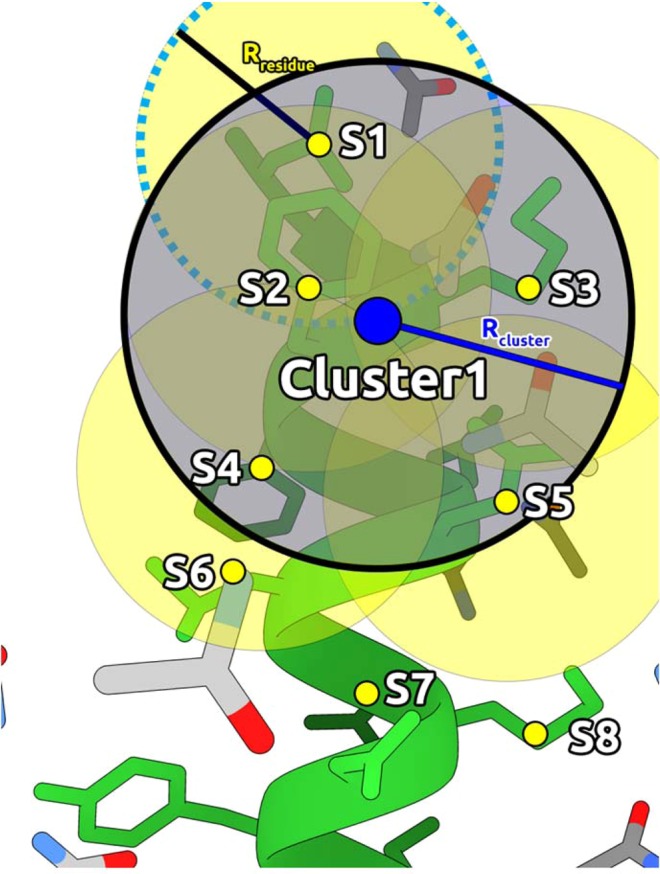
Clustering scheme of CAT: A sphere is generated per residue, which encapsulates shells of interacting cosolvent molecules (yellow circular regions defined by the variable R_residue_). Afterwards, a secondary clustering region (blue shaded area, defined by the variable R_cluster_) defines close side-chains centres of geometry, resulting in a series of representative clusters of interest.

To calculate the interaction scoring part per residue in the protein, CAT defines a sphere surrounding each residues centre of geometry (dashed blue circle in Fig. 1). Hence, the interaction energy between the protein and every probe inside the sphere is calculated. To avoid atomic clashes, softcore potentials^26^ were used, as described in Eq. 2.

2 $${E}_{i}={E}_{LJ}+{E}_{coulumb}=4\varepsilon [{(\frac{\sigma }{(r+{\delta }_{lj})})}^{12}-{(\frac{\sigma }{(r+{\delta }_{lj})})}^{6}]+\frac{K{q}_{i}{q}_{j}}{(r+{\delta }_{elec})}$$

where r is the interatomic distance, *ε* corresponds to the depth of the Lennard-Jones potential, *σ* is the finite distance to the zero potential, *K* is the Coulombic constant in kcal/mol, *δ*_*lj*_ and *δ*_*elec*_ are the softcore deltas for the Lennard-Jones potential and Coulombic potential respectively^26^.

With the assigned sphere, the average number of cosolvent molecules 〈*M*〉 inside can be calculated. S_interaction_ then can be calculated, in a simulation with N frames, as the ratio between the average interaction energy though the trajectory and the average number of molecules inside the sphere (Eq. 3):

3 $${S}_{interaction}=\frac{1}{\langle M\rangle }\mathop{\sum }\limits_{1}^{N}\frac{{E}_{i}}{N}$$

For the stability score, the RMSD of the total number of cosolvent molecules $\sqrt{\Delta {M}^{2}}$ inside the sphere was used in (Eq. 4):

4 $${S}_{stability}=\frac{(1-\sqrt{\Delta {M}^{2}})}{(\langle M\rangle -\sqrt{\Delta {M}^{2}})}$$

S_stability_ values range from 0 to 1, allowing the highest values for low variance, representing more stable interactions and molecules being retained for a longer time.

For the third scoring term, CAT counts the number of protein atoms (*J*_*contacts*_) inside each residue sphere (Fig. 1), assigning to it a volumetric score *S*_*depth*_. Afterwards, it is normalized by the highest scored residue, to set the range between 1 and 0, as Eq. 5 shows:

5 $${S}_{Depth}=\frac{\langle {J}_{Contacts}\rangle }{MAX\langle {J}_{Contacts}\rangle }$$

To define the regions, dummy atoms are created for every residue in its corresponding center of geometry, with its respective S_interaction_ (Eq. 1) assigned to it.

To define binding regions, CAT systematically scans through the protein backbone, defining a new spherical region (Fig. 1) which clusterizes the dummy atoms. This “CAT cluster” has a ${S}_{Region}$ assigned as (Eq. 6):

6 $${S}_{Region}=\,\frac{1}{{N}_{Residues}^{Inside}}\sum _{Inside}{S}_{Residue}$$

CAT generates a PDB file with dummy atoms highlighting the areas of interest regarding the S_residue_ per residue and S_Region_ per region, ranked from the best to worse. This is depicted in Fig. 1.

In this study, values for the electrostatic and Lennard-Jones softcore delta were scanned (Supplementary Information). The best result was attained with deltas set to 1 Å. The sphere radius for the residue–cosolvent interaction was set at 8 Å, to incorporate approximately 3 shells of solvation. The clustering sphere radius set at 5 Å, which encapsulates inter Cα distances for different secondary structures.

### Validation

In order to compare our results with other pocket detecting tools, all molecules studied in this work were also tested in the FTMap webserver, a well-known tool for the study of allosteric binding sites. FTMap method consists of accelerated molecular dynamics to calculate and equilibrate the structure, on which the surface is interacting with a series of probes. To determine whether FTMap has found the allosteric binding site, the probes shown in FTMapmust be interacting with residues which comprise the binding site of interest.

## Results

Driven by an increasing interest in identifying potential allosteric, transient, or cryptic sites for structure-based drug discovery (SBDD), Cosolvent Analysis Toolkit (CAT) has been developed to discover potential druggable hotspots from atomistic MD simulations. An in-depth study of the molecules used to test the accuracy of the scoring function and its corresponding ranking is done in this paper. The obtained results are directly compared to the FTMap webserver, a robust, powerful and widely popular ‘hotspot’ detecting tool. The comparison concludes that the explicit solvent/cosolvent interactions and MD sampling is crucial for the right assessment of cryptic binding sites, and CAT scoring function can filter and reasonably rank binding regions.

### Androgen receptor ligand binding domain (AR-LBD)

**Figure 2:**
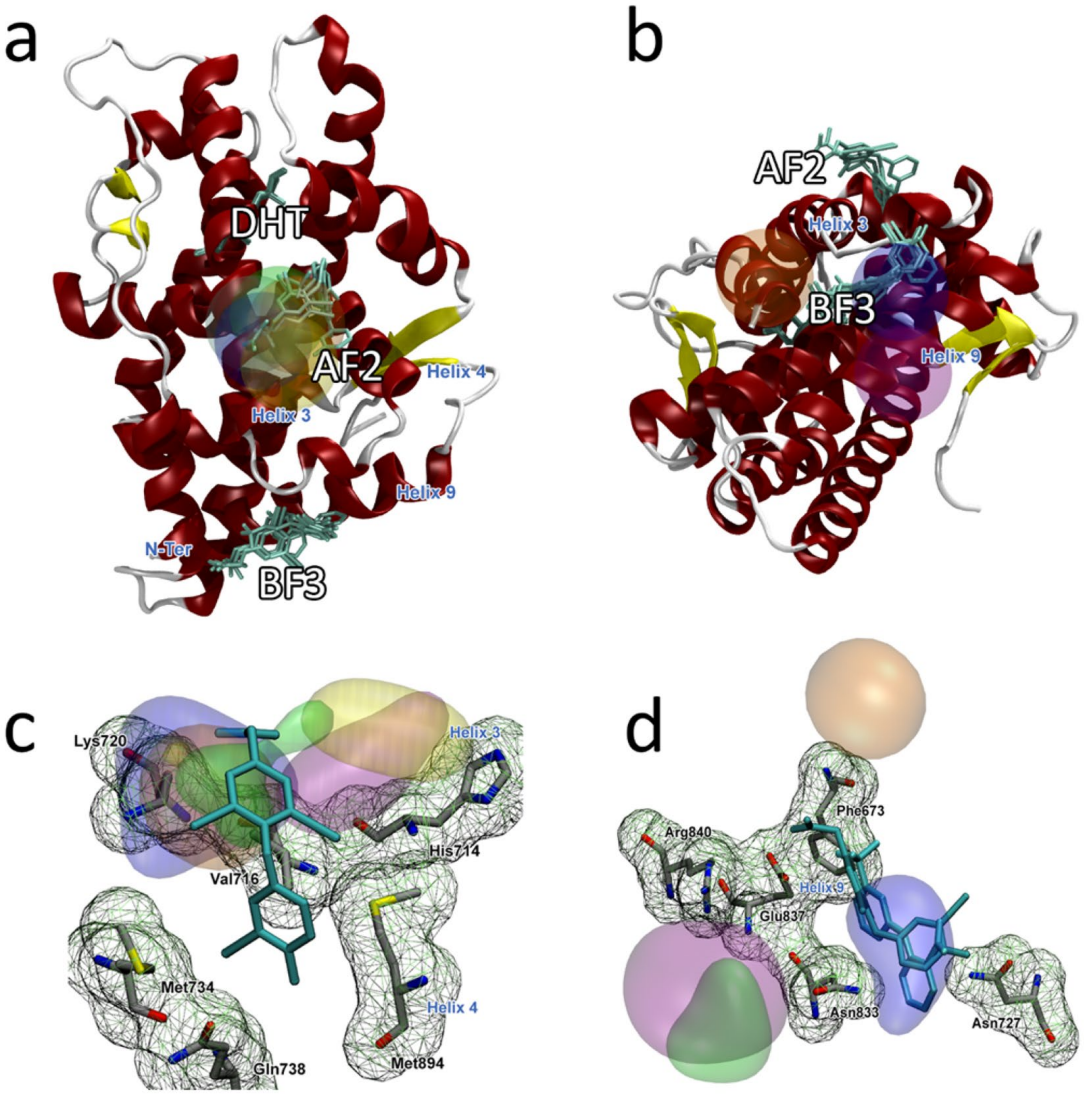
Androgen receptor LBD hotspots found by CAT. Clusters have the following colors assigned: acetamide – blue, benzene – purple, acetanilide – orange, imidazole – yellow, and isopropanol – green. The crystallographic ligand is colored cyan. (**a**) Panoramic representation of LBD domain centered on the AF-2 site compromised around the H3 and the respective top cluster given by CAT; (**b**) Panoramic representation centered around the BF-3 region and the respective CAT clusters. Simulations with all 5 probes found the site with a high rank, as described in Table 1. For the second site, only acetamide and benzene show high ranks. (**c**) AF-2 site and its key residues; K720, V716 and H714, that form part of H3, are detected by simulations with all 5 fragments. (**d**) BF-3 and its key residues; simulations with acetamide detected N833 and N727 as key residues for the site, but with a lower ranking than the clusters found in AF-2 site.

The androgen receptor is a multimeric DNA-binding transcription factor that regulates expression of genes critical for the development and maintenance of the male sexual phenotype. Through its ligand binding domain (LBD) it binds to steroid hormones such as testosterone, androsterone, or dihydrotestosterone; the binding event occurs at the orthosteric binding site. Furthermore, the presence of auxiliary allosteric binding sites has been reported in two of the solvent exposed regions of the protein: at the activation function 2 (AF-2) between helices 3 and 4 and at the binding function 3 (BF-3) close to helix 9 (Fig. 2).

The average structure with its respective CAT clusters have been superimposed to a series of experimentally-solved structures with bound ligands both in orthosteric and allosteric regions (Table 1)^28^. Binding poses of these ligands and their corresponding interactions with protein residues have been considered in the analysis. CAT finds both allosteric regions with fragments interacting with some of the key residues that achieve interactions with the crystallized ligands, as shown in Fig. 2. At the allosteric AF-2 binding site (Fig. 2), several highly-ranked clusters are placed mainly in helix 3(H3), including key residues K720 and V716 that present hydrophobic interactions with the ligand, as depicted Fig. 2c. This is consistent with the values of energy scores, as highest-ranked clusters in that area correspond to fragments with hydrophobic groups such as benzene and acetanilide. The more polar fragments also detect the H3 area but with a slightly lower cluster rank (Table 2). Nevertheless, these fragments are interacting with R726 and N727, two very flexible residues that can enclose or open the pocket. By visual inspection and covariance analysis (Supplementary Information), the shape of the pocket can considerably vary, given by the influence of these two residues, acting as the gatekeepers. Considering the small size of this binding site, the success of detection of this area as a potential ‘druggable’ hotspot is very encouraging. CAT identifies regions that not only interact with a couple of H3 residues, but with the majority of the residues within this site and surrounding sidechains that could contribute to the further pocket opening. The interaction of the cosolvents with helix 4 region is not as favorable as with other areas, as no highly-ranked clusters are found close to it.

**Table 2:** AR-LBD results and comparison with FTMap.

Target	Binding site	Protein contacts	Cosolvent	CAT rank	Found by FTMap?
AR-LBD	Orthosteric	E706, V746, R752, F764, H874, F878	Not found	—	✓
	AF-2 Allosteric	I672, F673, V716, K720, P723, G724, N727, K734	Acetamide	9	×
			Acetanilide	1,2,9	
			Benzene	1	
			Imidazole	5,7	
			Isopropanol	2	
	BF-3 Allosteric	F826, E829, Y834, E,833, R840, E897	Acetamide	1,3	×
			Acetanilide	6,7	
			Benzene	10	
			Isopropanol	8	

For the BF-3 allosteric binding site, CAT also gives satisfying results when compared with the experimental data. Although there is a higher number of CAT clusters in this area, especially around H9, the scoring rank of them is worse than the ones placed in AF-2. Key residues that give shape to the binding site, such as R840, Y834, G829 and F826, presented a CAT cluster, as Fig. 2d shows. In this case, acetamide is the fragment with most affinity to the area. Enclosing the site, CAT also detects interactions with the N-terminal area at F673 and the “gatekeeper” residues from AF-2 site: R726 and N727. To summarize, all clusters detected within AF-2 include the majority of the residues from this binding site. These residues are listed in Table 2.

The crystal structure of the AR dimer has been recently reported^40^, where the interactions between the AR monomers are observed. These interactions are crucial for the DNA binding and disrupting them could be a novel way to inhibit the protein. Interestingly, parts of the region involved in protein-protein interactions were detected by CAT along the dimerization interface. This highlights an applicability of CAT: mapping of novel and unique superficial interaction hotspots.

The only known AR binding site that CAT does not find is the orthosteric binding pocket. This site, which is a deep pocket binding dihydrotestosterone (DHT), is too enclosed inside the protein core and shielded from the surface for the cosolvent molecules to detect it. To observe the opening of this pocket it would require large conformational changes and thus simulations longer than performed in this study. It is very likely that in the timescales required some of the cosolvent molecules would undergo phase separation, therefore not appropriate for CAT analysis.

Comparison between CAT and FTMap shows some interesting results. FTMAP clearly identifies the orthosteric binding site as a potential ‘druggable’ hotspot (Table 2), as the highest populated clusters are mapped to that site. However, FTMAP fails to identify the allosteric binding sites: only one sparsely populated cluster is placed at the AF-2 site and none at the BF-3 site (Fig. 2). Moreover, unlike CAT, FTMap does not identify any dimer-forming regions of AR as potential hotspots. Therefore, an apparent strength of CAT is to reliably detect the hotspots that are challenging to FTMap.

### PTP1B

Tyrosine-protein phosphatase non-receptor type 1 (PTP1B) is a negative regulator of the insulin signaling pathway. It has emerged as a promising drug target for obesity and type II diabetes mellitus and obesity. Numerous potent PTP1B inhibitors have been discovered during last years, unfortunately nearly all medicinal chemistry efforts have been hampered by lack of selectivity and inhibition of related proteins, especially T-cell protein tyrosine phosphatase (TCPTP).

PTP1B orthosteric binding site is formed by three loops: the WPD with W179, P180 and D181, a phosphotyrosine (pTyr) loop including Y46, and a Q loop with G262^41^. An allosteric site (BB site) has been discovered by X-Ray crystallography, which has paved a new path to design selective PTP1B inhibitors. This site is located between helices 3 and 6, forming protein-ligand interactions with residues L192, A193, F196, E276 and F280. (Fig. 2)^42^. Furthermore, a series of binding events and allosteric sites have been identified by the means of multi-temperature crystallography, fragment screening, and covalent tethering. This last study includes more than hundred crystal structures and different binding events but for the sake of analysis, we will focus on the two newly tested and identified allosteric sites: the allosteric 197 site, close to the previously known BB allosteric site and the loop 16 (L16) site.

CAT analysis for the cosolvent MD simulations in the apo/open state (PDB code: 1XBO) identified both binding sites: orthosteric and allosteric. For the orthosteric site, all cosolvent molecules tested interacted with various regions of the site. As showed in Fig. 2a,b, imidazole mapped all regions of interest: WPD-, pTyr-, and Q- loops. Isopropanol interacted preferentially with the WPD loop, while acetamide, acetanilide, and benzene interacted with the pTyr loop residues. For the BB allosteric site, CAT placed clusters for all cosolvents except imidazole, with clusters centered at the binding site (Fig. 2c). Helix 3 was mapped in its entirety, as it was the helix 4 region that comprised the pocket along with its key residues. The close proximity of the 197 site to the BB site might mislead the analysis from CAT clusters as both pockets share residues. Although both pockets might be included for the same cluster the 197 site is mapped by CAT, mainly by acetanilide and benzene. Most of the clusters from this pocket included K197, the mutated residue in the work by Keedy and coworkers, to reassure the ‘druggability’ of this pocket^43^. Regarding the L16 site, CAT placed a series of highly ranked clusters close in the proximity of the binding site but in contact with just one, two or no pocket residues. Nevertheless, the level of mapping was sufficient enough to determine the area as a potential binding site region.

**Figure 3:**
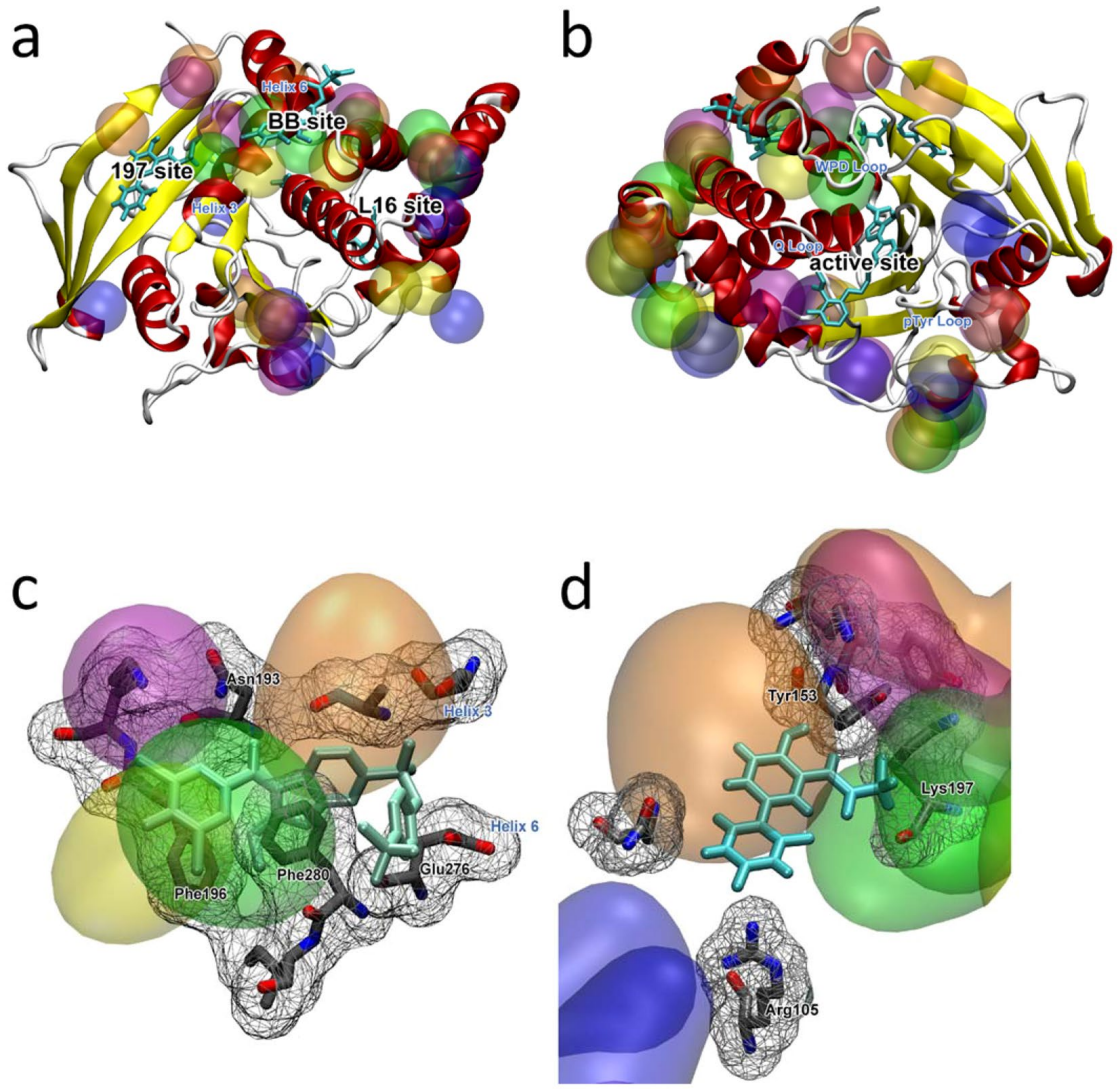
PTP1B hotspots found by CAT. Clusters have the following colors assigned: acetamide – blue, benzene – purple, acetanilide – orange, imidazole – yellow, and isopropanol – green. The crystallographic ligand is colored cyan. (**A**) Panoramic view centered on the allosteric binding sites; (**B**) View centered on the orthosteric binding site. CAT performs well finding and scoring the binding site for PTP1B since 4 out of the 5 cosolvent molecules are able to interact with the site residues. Only isopropanol and benzene find the orthosteric binding site, and acetamide interacts with neighbor key residues. (**C**) BB allosteric binding site and its main residues; all cosolvent molecules but acetamide rank clusters in the allosteric binding site, principally isopropanol, which shows interactions with N193, F196 and F280. (**D**) 197 site recently identified by Keedy *et al*.^43^. CAT maps the whole site, including K197.

FTMap has not identified the allosteric binding site (Table 3), which further emphasizes the strength of CAT in detection the allosteric hotspots that are difficult to find by FTMap. The comparison between CAT and FTMap shows a remarkable performance and robustness of the scoring function and the clustering method implemented in CAT. The drug-like small molecule bound at the allosteric PTP1B site reported by Wiesmann and coworkers using X-ray crystallography^42^ shows that this binding site is a bona fide ‘druggable’ site which can be used as starting point for a structure-guided design, which has been validated in the follow-up drug discovery efforts^42^. As shown in Fig. 3, CAT ranks the clusters at the orthosteric site high, yet it is not biased towards deep pockets, being able to report all experimentally detected pockets in the top-ranked 10 CAT clusters, including the allosteric site undetected by FTMap.

**Table 3:** PTP1B results and comparison to FTMap.

Target	Binding site	Protein contacts	Cosolvent	CAT rank	Found by FTMap?
PTP1B	Orthosteric	Y46, W179, P180, D181, G262	Acetamide	3	✓
			Benzene	5,6	
			Imidazole	6	
			Isopropanol	2	
	Allosteric	L192, A193, F196, E276, F280	Acetanilide	1,3,10	×
			Benzene	2	
			Imidazole	1,5,7	
			Isopropanol	3,6,7	
	197	R105, D148, K150, Y152, Y153, E157, N193, K197	Acetanilide	4,5,7	×
			Benzene	2,6	
			Isopropanol	5,6	
	L16	K237, K239, S242, I281	Acetanilide	1	×
			Benzene	1	
			Imidazole	10

### Fragment hotspot screening – GTPase HRas

**Figure 4:**
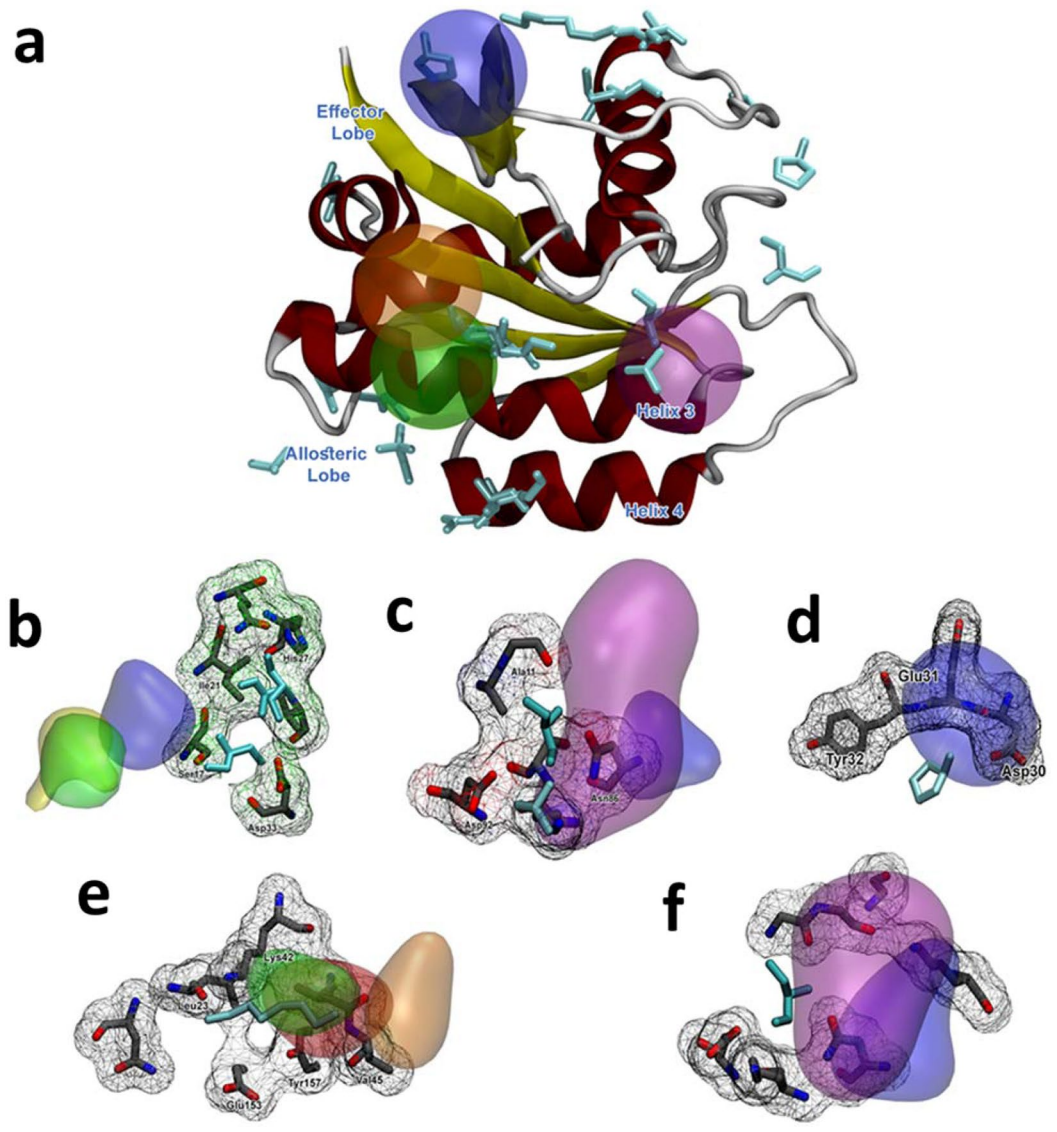
HRas hotspots found by CAT. The clusters are colored as follows: acetamide – blue, benzene – purple, acetanilide – orange, imidazole – yellow, and isopropanol – green. The crystallographic fragment is colored cyan. (**a**) Panoramic view of the HRas and the highest-ranked cluster for each cosolvent molecule. (**a**) Depiction of Site 3, (**b**) Site 5, (**c**) Site 6 (**d**) Site 7 and (**e**) Site 8, Following the naming and numbering from Buhrman *et al*.^49^. As shown, acetamide and benzene perform better than the other 3 cosolvent molecules, but the combination of the 5 different cosolvents are able to find most of the superficial binding sites and CAT score is able to find the interacting residues to different crystalized molecular fragments.

The main difference of the three isoforms of the human Ras proteins, HRas, KRas and NRas, lies within the primary sequence of the hypervariable region and its post-translational modifications^44^. The catalytic G-domains of the three respective Ras proteins are highly conserved, with only a 10% average difference in primary sequence identity in the C-terminal lobe (residues 87 to 171)^45^. The N-terminal lobe 1 carries the catalytic binding site with all the G-domains switches (Fig. 4)^46^.

The “effector lobe” contains the small molecule binding sites of Ras, including the allosteric site consisting of residues R97, D107 and Y137 (denoted as the allosteric lobe)^47^. This allosteric site is connected to the active site in HRas by helix 3 (H3), one edge of the inter-lobe linker, and one of the switches of the N-terminal lobe at the other. This is showed in Fig. 4.

Due to the sensitivity regarding the conformational changes of the HRas, the cosolvent MD simulations prior to the CAT analysis were run only in the “off” state, to enable the direct comparison with the experimental MSCS (Multiple Solvent Crystal Structure) results on the H-Ras^8,48^. The MSCS showed several hotspots formed in different regions of the protein in the “off” conformation. The CAT analysis shows that our method can detect several of these hotspots in highly-ranked clusters.

Two major ‘hotspots’ were identified for HRas: one found in the inter-lobe linker region, and another one in the allosteric lobe (Fig. 4). Both hotspots involve H3 helix, but each of them is situated on either side of the helix. Cluster 1, as numbered in the study by Buhrman *et al*.^49^, is located near to the active site, between H3 and switch II, showing R68 and Y96 as the major contributors. Several highly-ranked CAT clusters interacted with cluster 1 residues, mainly in helix H3. All fragments but acetamide interacted with the key residues R68 and Y96. Although acetamide did not interact with these amino acids, it placed its highest-ranking cluster around a large region of H3. Cluster 2, found between helices H3 and H4, mapped to one of the largest hotspots. In this case, CAT interacted with both helix 3 and 4, with residues I93 and H94 from helix 3 and virtually all residues from helix 4. There were no acetamide clusters found around the pocket, which may indicate that this region has a low affinity for highly polar moieties. Cluster 4 consisted of a pocket in the inter-lobe linker region very close to the nucleotide substrate. It was comprised of D30 and K147; the latter being a target for ubiquitination on Ras-GTP^50^. There were only two CAT clusters that interacted with the residues from this pocket. Acetamide interacted with D30, while imidazole did with K147. Remaining clusters mapped to the pockets that overlapped with sites occupied by effector Ras binding (RBD) or cysteine-rich (CRD) domains and RasGAP.

At the inter-lobe linker region and at the region overlapping with Raf-CRD, CAT has mapped cluster 7. Clusters 3 and 6 overlapped with the RasGAP binding site. Although CAT mapped all experimentally detected sites, its performance for the lower-ranking clusters was worse than for the first two hotspots. Not all the cosolvents interacted with these binding sites. Interestingly, binding sites mapped by MD simulations using our most polar cosolvent, acetamide, did not overlap with hotspots detected by other fragments (and *vice versa*). This may imply that putative hotspots detected by acetamide might not be druggable, or that they may be very small hence not amenable for fragment growth and structure-based ligand design.

FTMap detected only hotspots marked by clusters 1 and 2; both being among highly-ranked FTMap clusters. On the other hand, FTMap detected the calcium acetate binding site^51^ whereas neither CAT nor MSCS succeeded (Table 4).

**Table 4:** HRas results and comparison to FTMap.

Target	Binding site	Protein contacts	Cosolvent	CAT rank	Found by FTMap?
HRAS	Site 1	R68, Q95, Y96, Q99, D92	Acetamide	1	✓
			Acetanilide	2,7	
			Benzene	1,5	
			Imidazole	1,4,5	
			Isopropanol	2,4	
	Site 2	H94, L133, S136, Y137	Acetanilide	1,5,9	✓
			Benzene	8	
			Imidazole	4,7,10	
			Isopropanol	2,5,6	
	Site 3	S17, I21, Q,25, H27, V29, D33, T35, D38, Y40	Acetamide	3,9	×
			Imidazole	8	
	Site 4	F28, D30, K147	Acetamide	3	×
			Imidazole	9	
	Site 5	A11, G12, N86, K88, S89, D92	Acetanilide	9	×
			Benzene	3	
			Imidazole	4	
			Isopropanol	5,6	
	Site 6	D30, E31, Y32	Acetamide	3	×
	Site 7	L23, N26, K42, V44, V45, R149, E153, Y157	Acetanilide	6,8	×
			Benzene	4,6	
			Isopropanol	7	
	Site 8	G13, Y32, N86, K117	Acetamide	3	×
			Benzene	2	

### Novel allosteric sites prediction on CDK2

Cyclin-dependent kinase 2 (CDK2) is a serine/threonine ATP-binding kinase that interacts with several different cyclins^52^. It is comprised of two regions known as C and N lobes, connected by a hinge, with a significant role in the cell cycle, in the transcription regulation^53^. CDK2 directly acts on the protein expression related to the transition from the G1 to S phase of the cell cycle. Hence, it is an interesting protein target for cancer drugs. Functionally, CDK2 goes through a series of conformational changes to reach an active state. The interlobe region interacts with cyclins (preferably A and E), shifting the activation loop (located between residues A149 to T165) and subsequently revealing the ATP binding site. This allows the phosphorylation of the threonine located in the active site, reaching a final active configuration.

Recently, CDK2 was used in a novel experimental approach for the identification of binding sites called Fraglite. Wood and coworkers experimentally mapped a series of allosteric sites using X-ray crystallography, resulting in a set of 5 regions with known fragment binding. One of the main features in these set of structures is the fragments were designed to achieve hydrogen bonds, improving the assessment on its allosteric druggability and tractability.

To further assess the capacities of the CAT scoring function, cosolvent runs were made with all five previously cited probes. Since most of these sites are recently discovered, it should result in an evidence of non-biasing of our described energetic scoring function. Hence, CAT is able to rank all 5 novel fragment binding sites described in Wood *et al*. along with the ATP binding site. Description of found hotspots is shown in Table 5.

**Table 5:** CDK2 results and comparison to FTMap

Target	Binding site	Protein contacts	Cosolvent	CAT rank	Found by FTMap?
CDK2	Orthosteric	E12, G13, Q131, N132, D86, L134, L134, D145	Acetamide	1	✓
			Imidazole	4,8	
	Site 1	K33, K34, Y77, K6, Y19, L32, K75, K34, H71L	Acetanilide	1,9	×
			Benzene	1,3	
			Isopropanol	2,3	
	Site 2	T160, H161, R157, T158	Acetamide	7	×
			Imidazole	6	
			Benzene	5	
			Isopropanol	4	
	Site 3	L124, R150, G147, H125, R126, C177, J178, Y179	Acetamide	3	✓
			Acetanilide	2, 4, 5	
			Benzene	5, 8	
			Imidazole	9	
			Isopropanol	8	
	Site 4	T221, P222, D223, L219, R245, L267, Y262	Acetanilide	2, 4	×
			Imidazole	10	
	Site 5	R199, T198, M192, T97, I104	Acetamide	3	×
			Acetanilide	2, 8, 10	
			Isopropanol	7	

**Figure 5:**
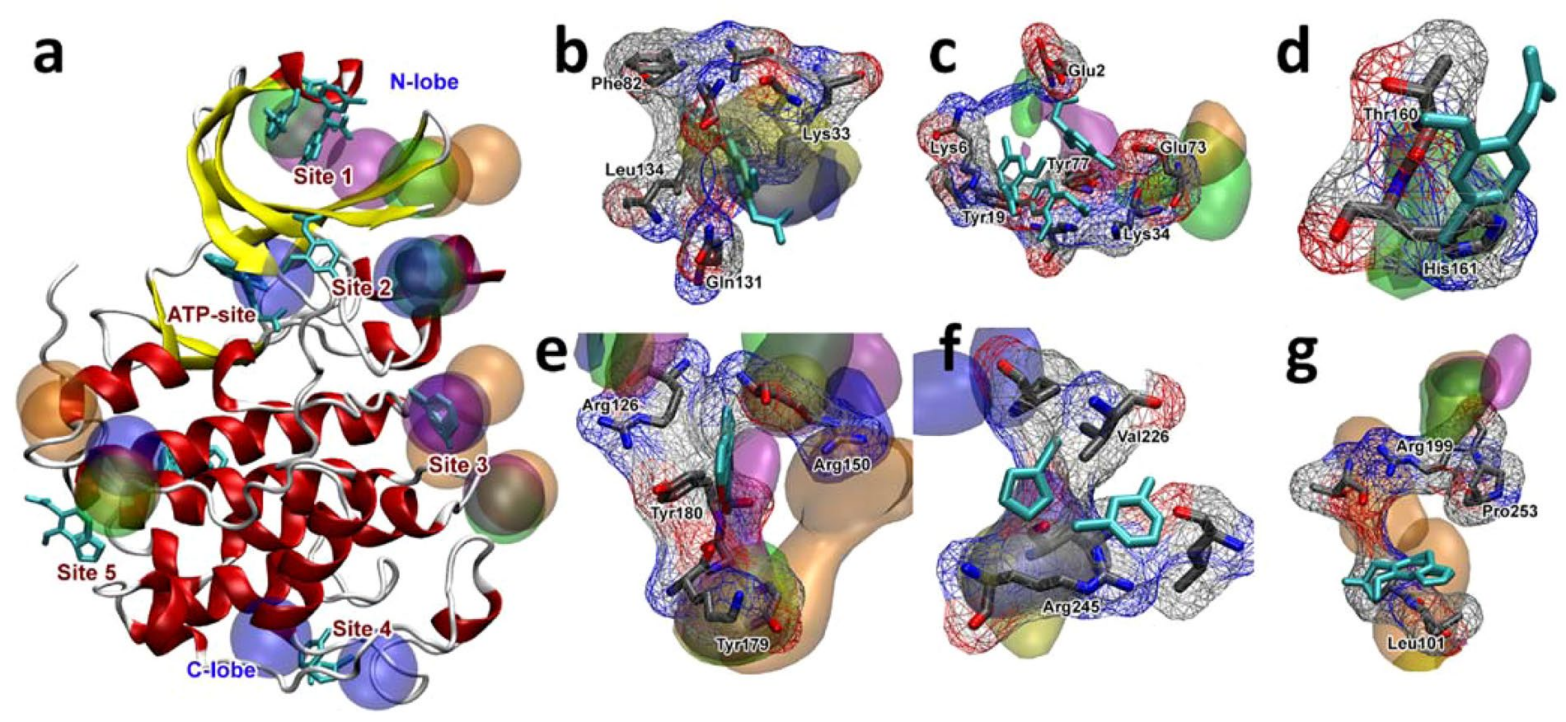
CDK2 Hotspots found by CAT. The clusters are colored as follows: acetamide – blue, benzene – purple, acetanilide – orange, imidazole – yellow, and isopropanol – green. The crystallographic fragment is colored cyan. (**a**) Panoramic view of the HRas and the highest ranked cluster for each cosolvent molecule. (**a**) Depiction of CDK2 and highest scored clusters, (**b**) Orthosteric site, (**c**) Site 1 (**d**) Site 2 (**e**) Site 3 (**f**) Site 4 (**g**) Site 5. As shown, acetamide and acetanilide perform better than the other 3 cosolvent molecules, given the nature of the experimental X-ray mapped crystallographic binding regions. Site 4 and 5 in specific shows high ranked clusters for these 2 probes, given by the high polarity of the site’s side chains.

Site 2 (Fig. 5) is located exactly on the activation loop. CAT was able to highlight all residues comprising this loop, including T160, with overlapping clusters of several different cosolvents. This threonine goes through a phosphorylation event, being one of the main contributors of binding site stabilization. The region along site 3 represents the dimerization area where the binding of cyclin partners occurs, being found by CAT with different cluster ranks. Protein-protein areas are commonly highlighted by CAT scoring function, given the calculated energetic aspect which can filter highly favorable interactions within shorter residence periods of cosolvent molecules.

Sites 4 and 5 (Fig. 5) are located in the C-Lobe region. Site 4 is directly related to the C-lobe loops and it is a novel binding site for CDK2. It interacts with polar residues (such as T221 and R245), which explains its high affinity with acetanilide. This pocket represents areas that, when constrained, could change the dynamics of the semi-unstructured T221-D247 C-Lobe loop, which is related to the cyclin dimerization stabilization event, resulting in an interesting site for structured based drug design. Site 5 is found at the end of the α-helix bundle that comprises most of the C-lobe sequence. It is highly ranked in CAT, especially for cosolvents highly polar probes, such as acetamide and acetanilide. As described in Wood and coworkers^29^, fragments using in their study should be tailored to accurately find a specific binding region by the usage of fragments prone to form hydrogen interactions. Hence, the used structures should represent highly specific interactions, resonating with the results given by CAT, ranking polar molecules in the same manner.

## Discussion

The correct assessment of the structural changes within the protein target is crucial for the right evaluation of possible time-dependent binding sites. As such, an accurate tool is pivotal for selecting possible contact regions to be further studied. While standard analysis of hotspot mapping quantifies primarily the volume of the binding region, cosolvent MD simulation followed by CAT analysis focuses on the cosolvent-induced conformational changes, to map, assess, and rank the putative ‘hotspots’, via an empirical scoring function. This characteristic gives the algorithm presented herein a high level of robustness and reliability in searching and ranking hotspots, as shown by the comparison with experimental data and FTMap predictions. The scoring function implemented in CAT makes it unique and distinct from computational methodologies reported in the literature.

Upon inspection of the androgen receptor ligand binding domain (AR-LBD), CAT outperformed FTMap, by finding both allosteric binding site from cosolvent MD simulations data employing several different cosolvent molecules. CAT analysis found, on average, 70 clusters mapping to putative interacting regions aka ‘hotspots’, and it was able to correctly rank the clusters which represented both of the binding sites in the top 10 clusters (Table 2 and Supp. Table 1). FTMap failed to find known AR-LBD allosteric sites. This demonstrates the robustness of the framework behind the scoring function implemented in CAT: unlike the scoring method used in FTMap, CAT does not involve any knowledge-based potential, thus not having any comparative bias regarding known binding modes.

Similar performance has been observed for PTP1B: CAT has successfully mapped and correctly ranked the allosteric binding sites of PTP1B along with its orthosteric site; a task that FTMap was unable to accomplish. The BB allosteric binding site of PTP1B is prone to undergo conformational changes upon the formation of the orthosteric complex and although both 197 site and L16 sites have been recently identified and it has been determined their allosteric role. The protein in the cosolvent environment is able to bind cosolvent molecules to its orthosteric site, which facilitates the opening of the allosteric binding site. This allosteric communication cannot be probed using the FTMap methodology, which does not take into the account changes in correlated motions upon the binding event. The H-Ras case further tested the robustness of CAT and its predictive power towards novel molecular hotspots. As shown by Burhman *et al*., FTMap was able to find all putative binding sites validated by MSCS but failed to find a series of hotspots found in the crystal structures. CAT, on the other hand, was able to find 7 out of the 8 crystallographically identified binding sites. It should be emphasized, however, that all sites identified by MSCS are shallow and very small, and very likely these are too small to be considered as bona fide ‘druggable’ binding sites.

CDK2 novel sites were correctly mapped and ranked by CAT. The ATP binding site is found in the acetamide runs as the top cluster. This differentiates from the previous tests, which were unable to find deeper sites, showing that CAT is also able to find deeper regions of interest. For the allosteric sites, CAT was able to map all five novel binding regions. Two sites are directly related to the cyclin-CDK dimerization interface (sites 2 and 3). Site 3 in specific is in direct interaction with T160, but might not represent a ‘druggable’ region given its superficiality.

CAT is a tool that eases the analysis of cosolvent MD simulations, reducing the manual observation of every trajectory to a set of clusters ranked through a scoring function based on energy and geometrical parameters.

CAT is not the first cosolvent tool in the literature, and it might still show some flaws compared to its predecessors, like for example the identification of androgen receptor’s deep-buried orthosteric binding site. CAT ease of use and complementarity with most popular visualization tools for current users is what makes it a really orthogonal option. Alongside that, CAT was built in a readily customizable way, to accepted auxiliary parameters. Thus, different force fields or charge models can be easily imported and used. Furthermore, the use of a scoring function based on both geometrical and energy values could additionally help future structure-based drug design such as identification of pockets key residues or design of pharmacophore models.

It is interesting to remark the recent development of a Pymol plugin based on the MixMD approach called ProbeView. With a similar idea to CAT, Probeview helps deepen the understanding of cosolvent probing for allosteric binding sites, but it is restrained to Pymol. A significance is on visualization: the fact that a CAT output can be seen in any visualization tool, not only Pymol, can make the usability higher. Probeview, used alongside CAT, may represent a useful orthogonal scoring methodology to assess a diverse range of binding sites.

The scoring function implemented in CAT can successfully filter and rank hotspots in a dynamical environment provided by cosolvent MD simulations. In the present study, we have developed, tested and validated the applicability of CAT analysis to detect several potentially ‘druggable’ allosteric sites, which were detected by X-Ray crystallography studies. The usage of five different cosolvent molecules demonstrated, at the same time, a broad sample space regarding interacting molecules and provides an insight on the chemical nature of the putative ligand moieties that would preferentially bind to the respective site. CAT is robust yet versatile: the analysis can be performed on cosolvent trajectories using any cosolvent molecule of choice.

The major shortcoming of CAT observed so far was its inability to map some deep buried pockets. This could be attributed to insufficient sampling during MD simulation, however, FTMap performs very well on this task. Although this issue may be easily sorted by longer MD simulation in water prior to cosolvent MD simulations, a combination of both tools could be a viable approach. We understand that the principle in which FTMap is based is not the same, although they share some features. Our main goal while choosing this tool as a comparison with CAT relied on the ease of use and fast results one could get. The use of cosolvent tools such as CAT can give more insights into the dynamics and “crypticity” of the target in comparison to FTMap.

In future works, we aim to explore CAT analysis applied to multi-cosolvent trajectories and to address the sampling problem which underlies the sub-optimal performance in mapping the buried pockets.

## Supplementary information

Supplementary Information

## Data Availability

The authors declare that all the data supporting the findings of this study are available within the article and the Supplementary Information, and upon request. The CAT source code, tutorial and respective test files can be found in https://github.com/ammvitor/CAT.
