# Supplementary material for: Cosolvent Analysis Toolkit (CAT): a robust hotspot identification platform for cosolvent simulations of proteins to expand the druggable proteome
Source: Sci Rep. 2019 Dec 13;9:19118. doi: 10.1038/s41598-019-55394-2 (PMC6910964; doi:10.1038/s41598-019-55394-2)
Supplement: Supplementary file 1 — Supplementary Information [file 41598_2019_55394_MOESM1_ESM.pdf]

Supplementary Information

Cosolvent Analysis Toolkit (CAT): a robust hotspot identification platform for cosolvent simulations of proteins to expand the druggable proteome

Francesc Sabanés Zariquiey*, João V. de Souza*, Agnieszka K. Bronowska

Chemistry – School of Natural and Environmental Sciences, Newcastle University, NE1 7RU Newcastle, United Kingdom

*equal contribution

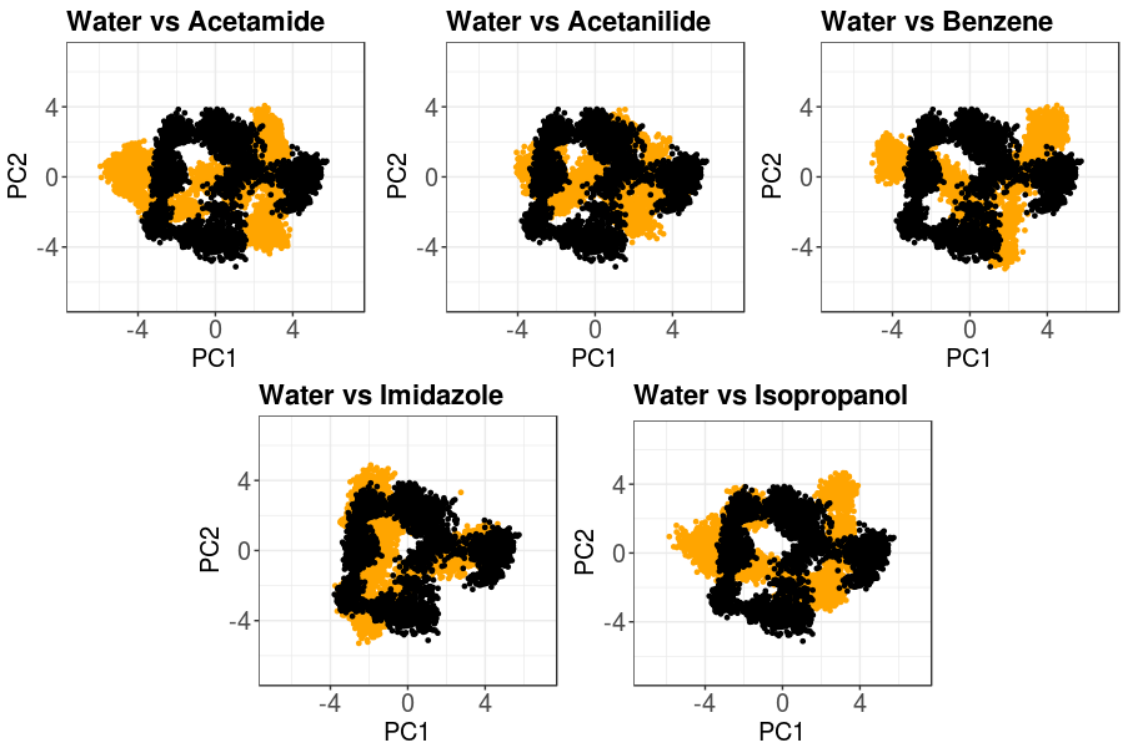

Supporting Figure 1: PCA distribution of control simulations (Protein-Water) with cosolvent systems in HRAS GTPase. Black dots correspond to the control simulation and orange dots to the cosolvent MD.

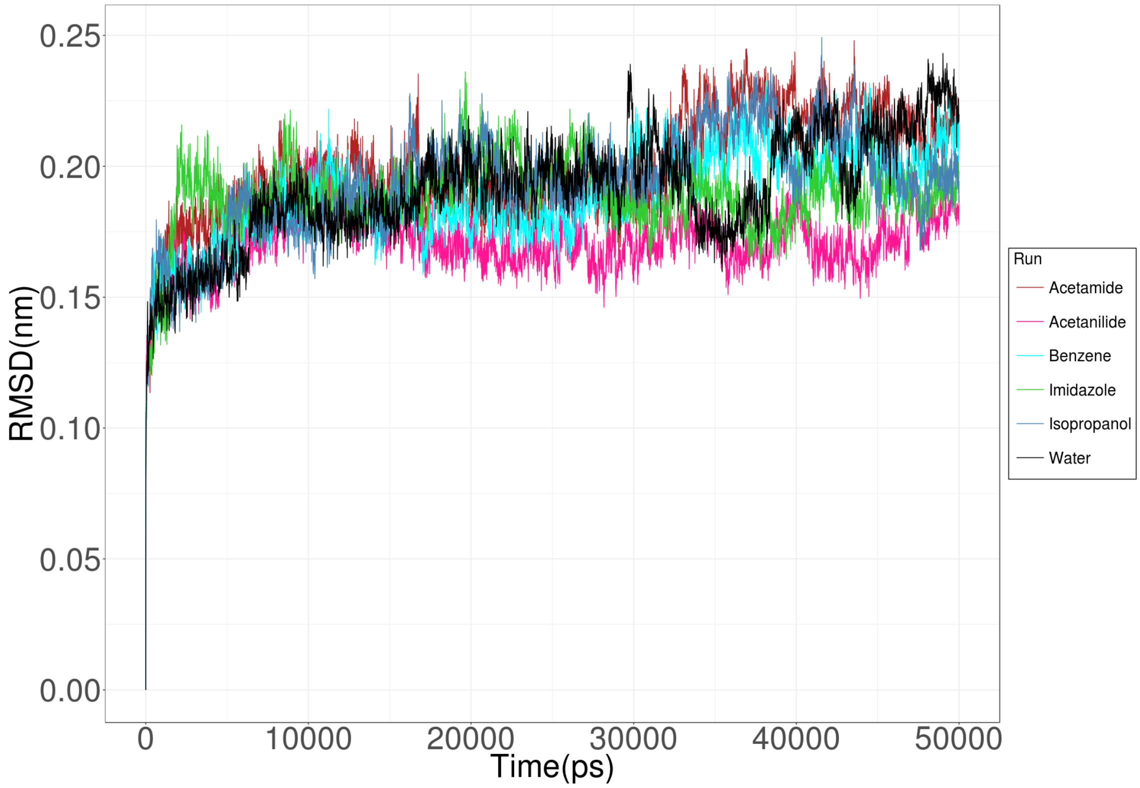

Supporting Figure 2: RMSD plots for HRAS 5 cosolvent runs and water simulation.

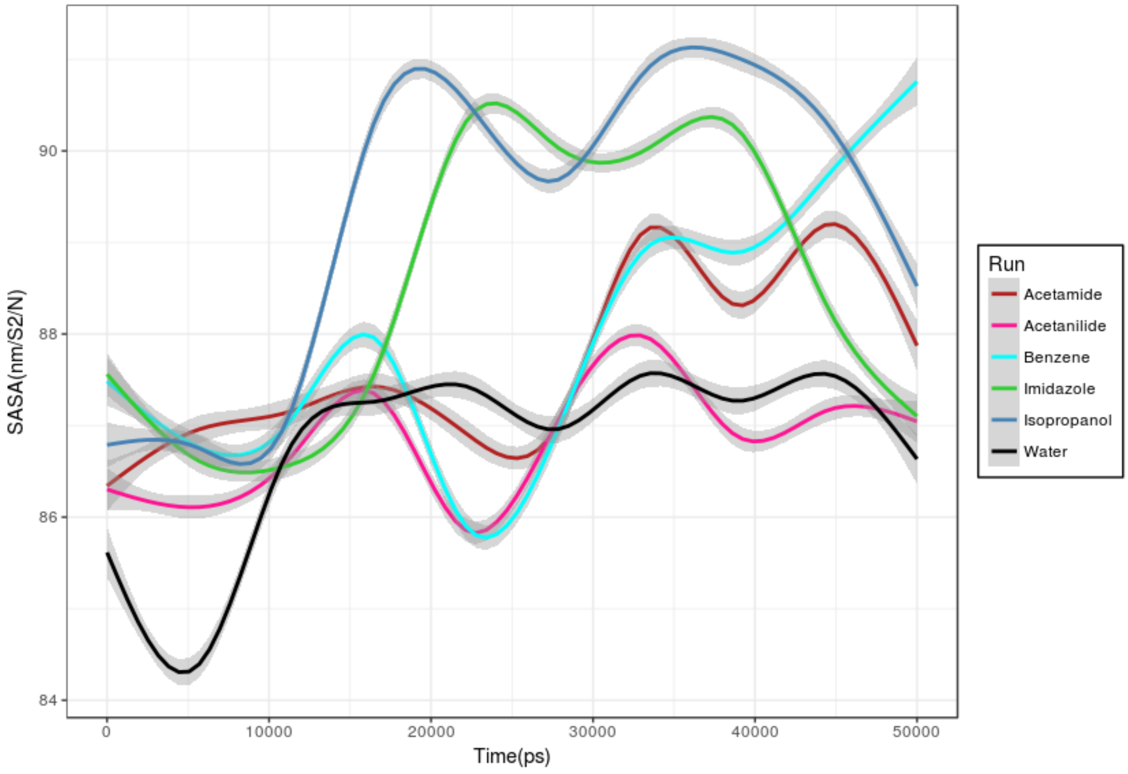

Supporting Figure 3: SASA plots for HRAS 5 cosolvent runs and water simulation.

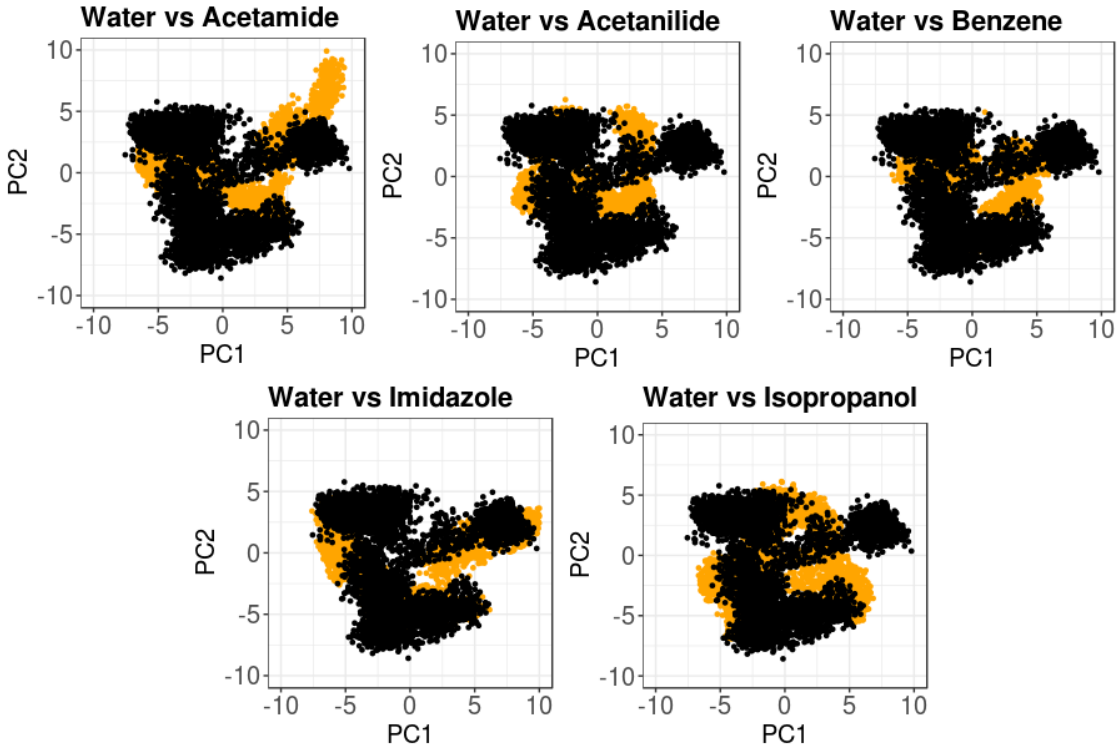

Supporting Figure 4: PCA distribution of control simulations (Protein-Water) with cosolvent systems in PTP1B. Black dots correspond to the control simulation and orange dots to the cosolvent MD.

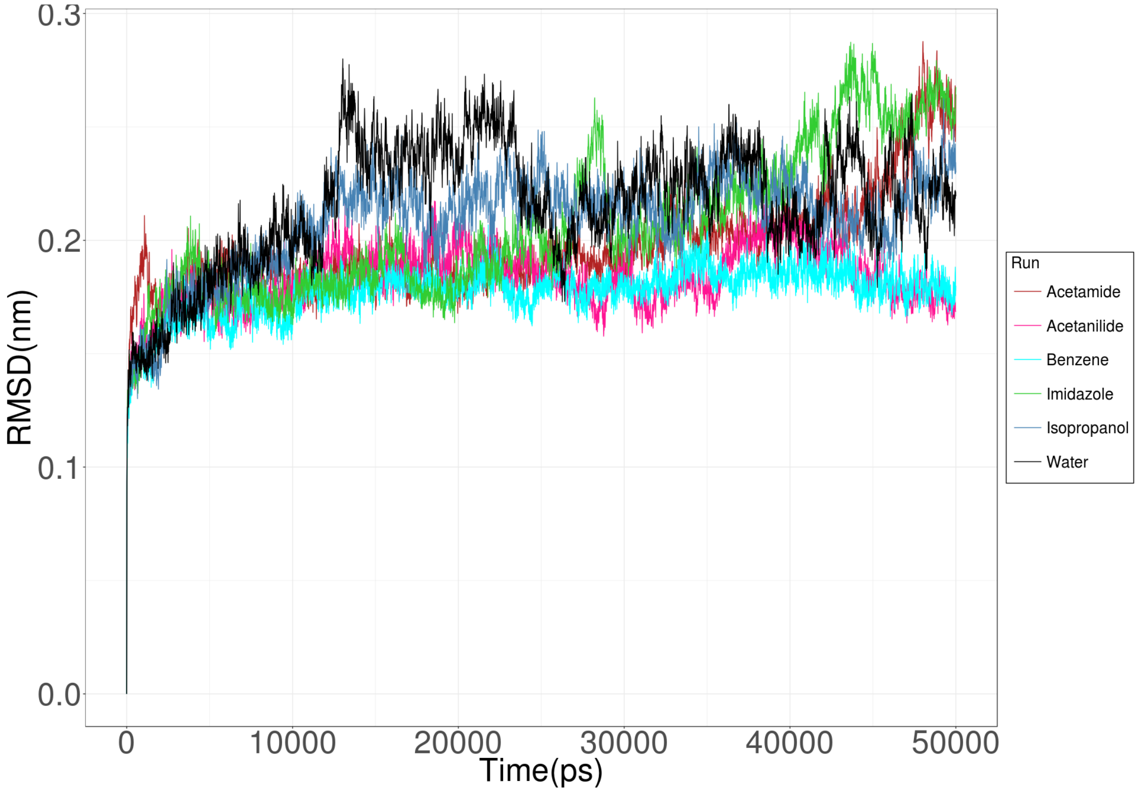

Supporting Figure 5: RMSD plots for PTP1B for all 5 cosolvent runs and water simulation.

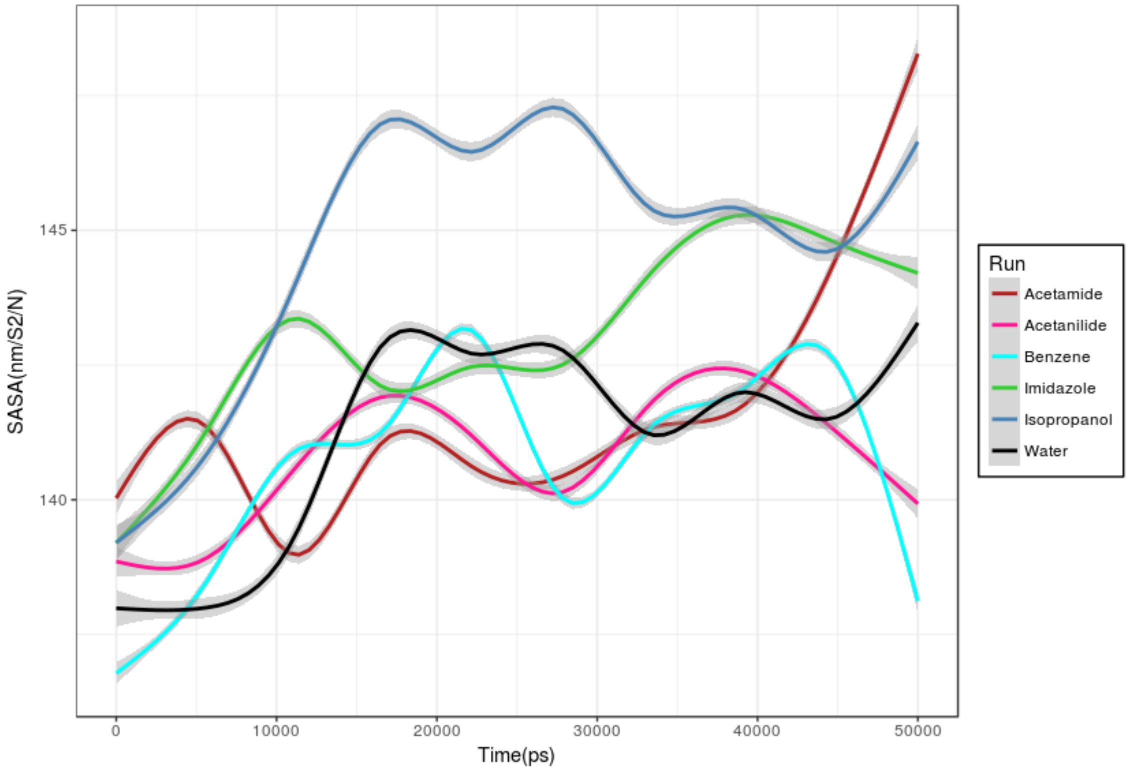

Supporting Figure 6: SASA plots for HRAS 5 cosolvent runs and water simulation.

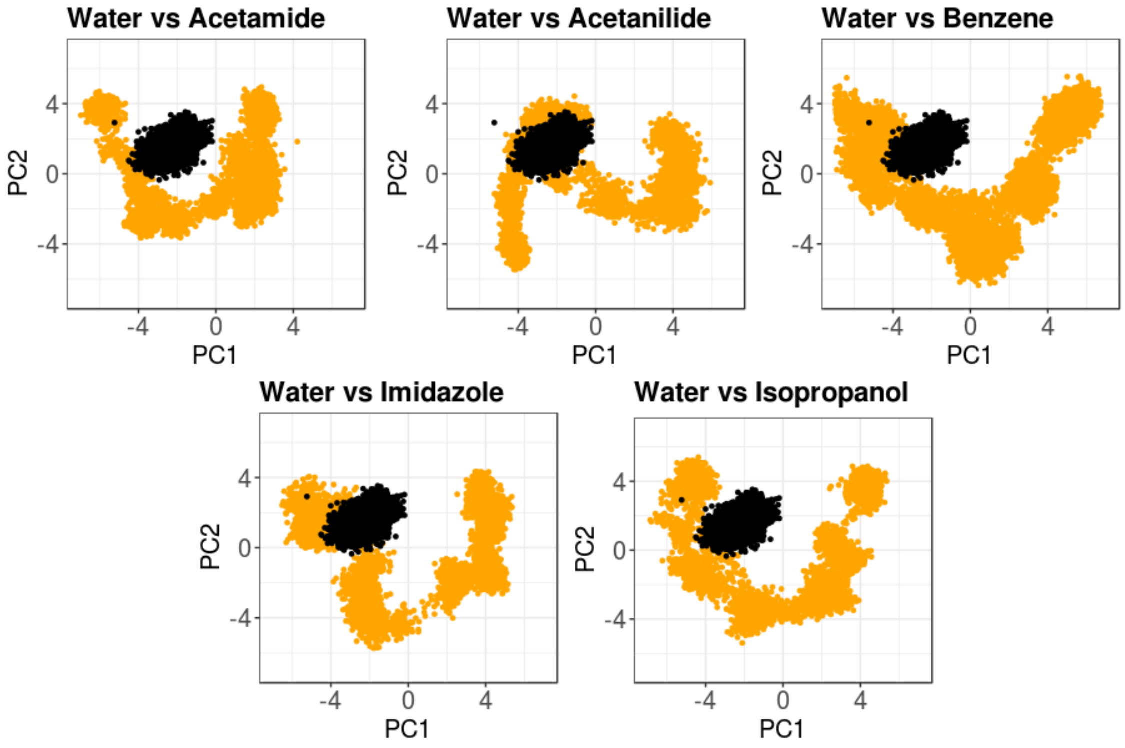

Supporting Figure 7: PCA distribution of control simulations (Protein-Water) with cosolvent systems in AR ligand-binding domain. Black dots correspond to the control simulation and orange dots to the cosolvent MD.

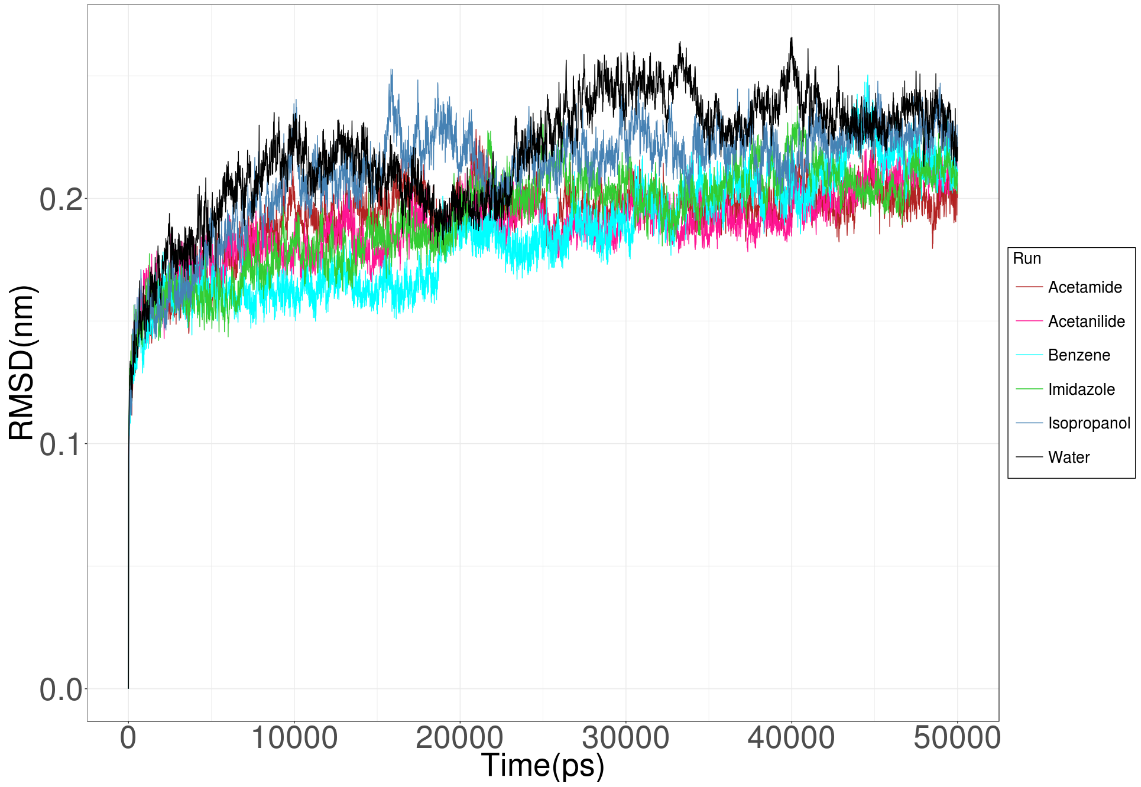

Supporting Figure 8: RMSD plots for AR-LBD cosolvent runs and water simulation.

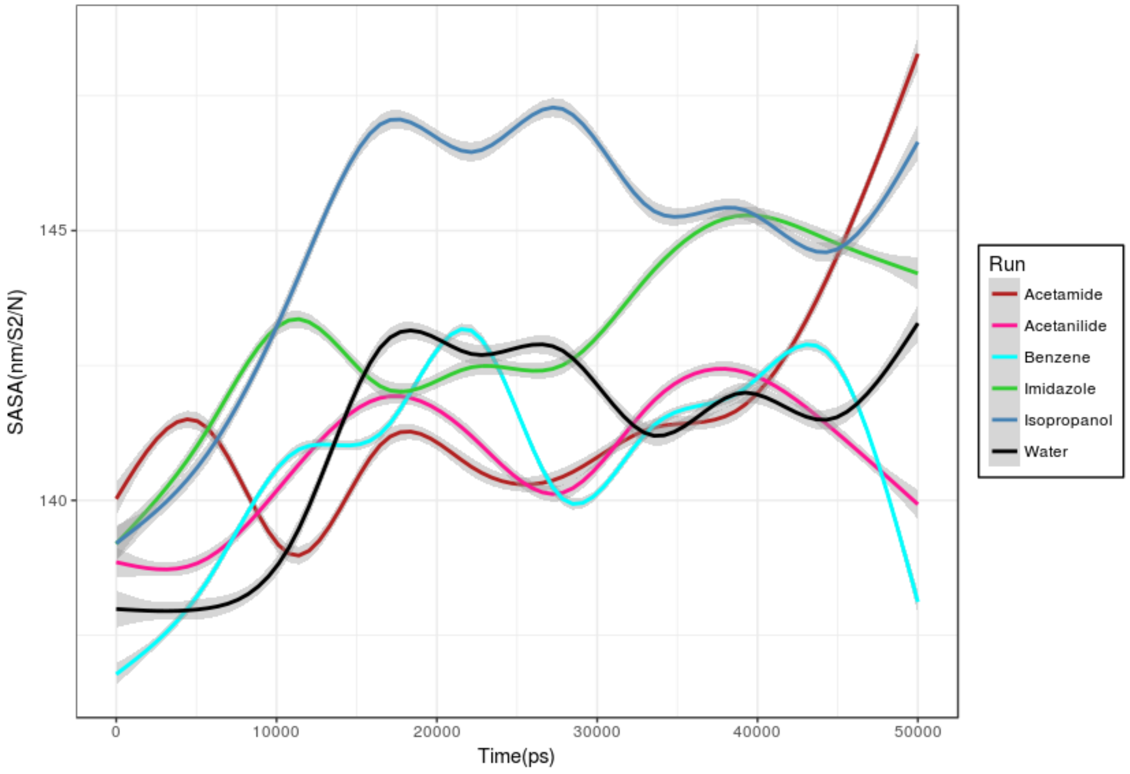

Supporting Figure 9: RMSD plots for AR-LBD cosolvent runs and water simulation.

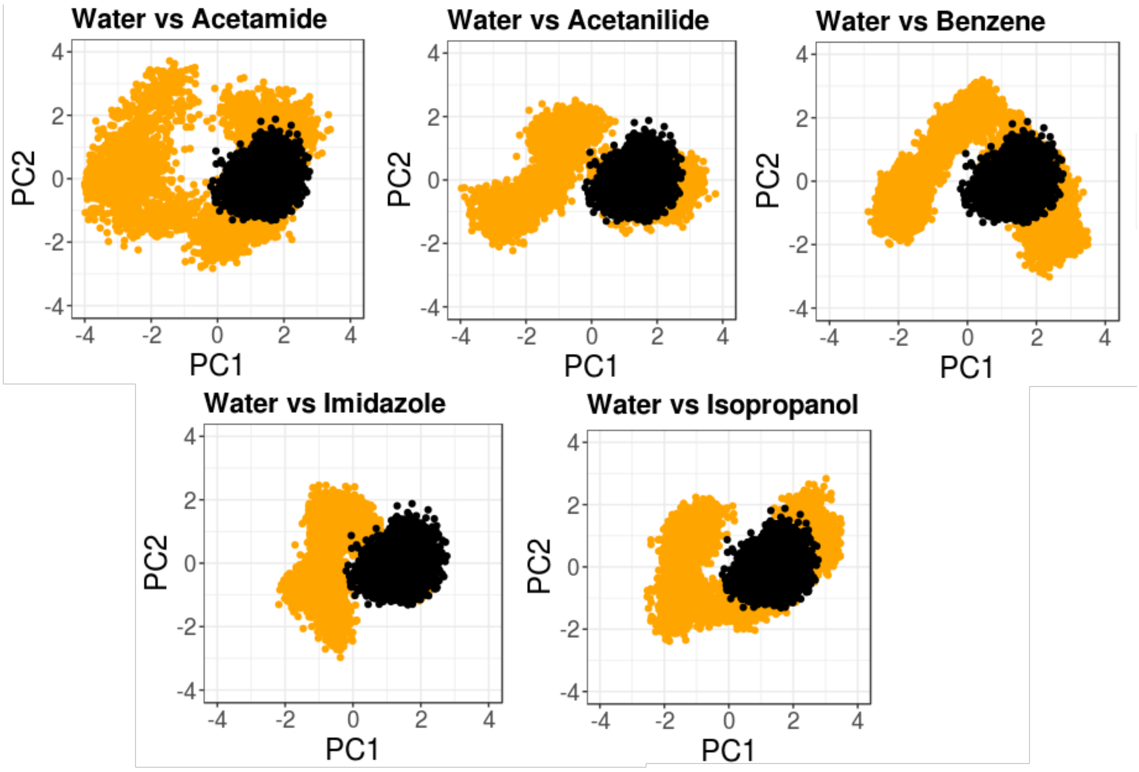

Supporting Figure 10: PCA distribution of control simulations (Protein-Water) with cosolvent systems for CDK2. Black dots correspond to the control simulation and orange dots to the cosolvent MD.

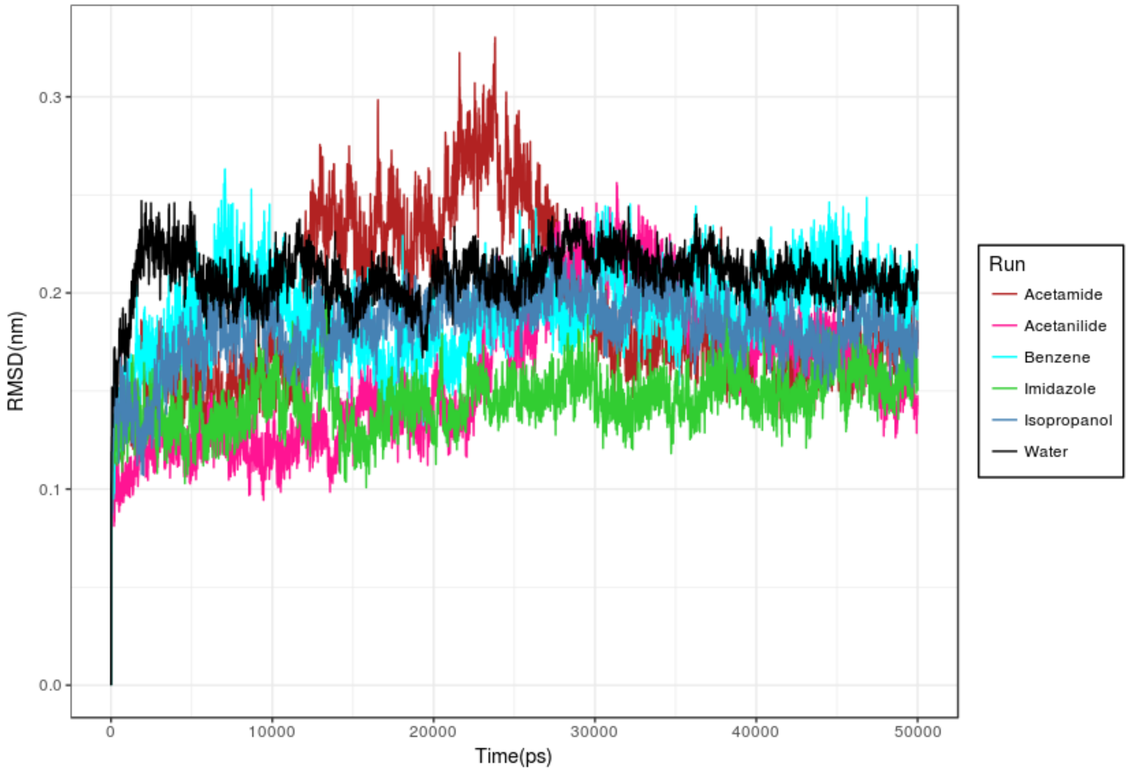

Supporting Figure 11: RMSD plots for CDK2 cosolvent runs and water simulation.

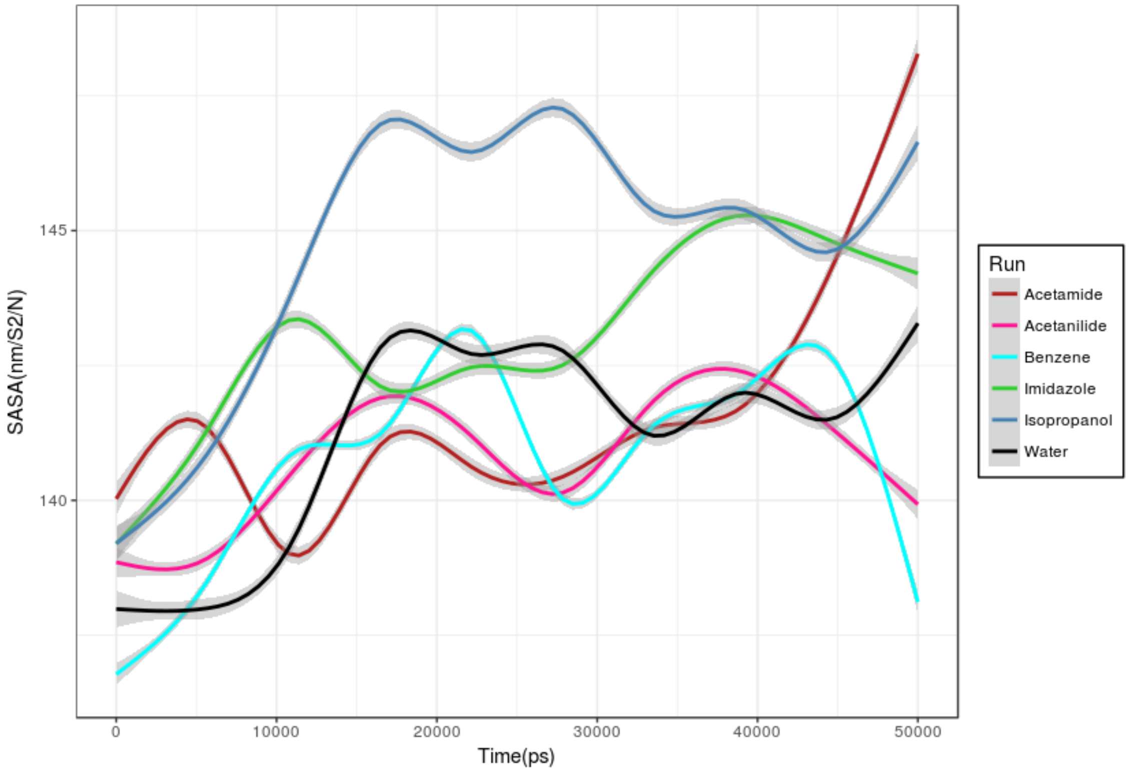

Supporting Figure 12: SASA plots for CDK2 5 cosolvent runs and water simulation.

Supporting Table 1: CAT scores for all targets and the top 10 clusters

AR-LBD					
**Rank/Cosolvent**	**Acetamide**	**Benzene**	**Isopropanol**	**Acetanilide**	**Imidazole**

1	-0,65	-0,68	-0,93	-1,15	-1,73
2	-0,61	-0,64	-0,84	-0,98	-1,49
3	-0,56	-0,54	-0,81	-0,83	-1,29
4	-0,51	-0,45	-0,71	-0,74	-1,13
5	-0,46	-0,35	-0,68	-0,62	-1,02
6	-0,43	-0,35	-0,62	-0,54	-0,94
7	-0,43	-0,33	-0,56	-0,54	-0,87
8	-0,41	-0,27	-0,5	-0,49	-0,84
9	-0,41	-0,24	-0,42	-0,47	-0,82
10	-0,4	-0,24	-0,42	-0,45	-0,81
**PTP1B**					
**Rank/Cosolvent**	**Acetamide**	**Benzene**	**Isopropanol**	**Acetanilide**	**Imidazole**
1	-2,3	-0,47	-1,65	-0,88	-1,68
2	-1,08	-0,47	-0,92	-0,53	-1,02
3	-0,8	-0,42	-0,58	-0,44	-0,78
4	-0,68	-0,31	-0,57	-0,4	-0,77
5	-0,67	-0,3	-0,49	-0,4	-0,74
6	-0,54	-0,28	-0,43	-0,34	-0,73
7	-0,53	-0,26	-0,42	-0,29	-0,65
8	-0,52	-0,24	-0,42	-0,29	-0,62
9	-0,51	-0,22	-0,41	-0,29	-0,58
10	-0,51	-0,22	-0,4	-0,28	-0,57
**HRas**					
**Rank/Cosolvent**	**Acetamide**	**Benzene**	**Acetanilide**	**Imidazole**	**Isopropanol**
1	-0,64	-0,32	-0,4	-0,83	-0,55
2	-0,64	-0,19	-0,33	-0,64	-0,5
3	-0,57	-0,17	-0,3	-0,59	-0,49
4	-0,52	-0,17	-0,28	-0,57	-0,49
5	-0,52	-0,16	-0,22	-0,56	-0,42
6	-0,45	-0,15	-0,21	-0,51	-0,41
7	-0,43	-0,15	-0,2	-0,49	-0,41
8	-0,39	-0,14	-0,19	-0,45	-0,34
9	-0,37	-0,14	-0,19	-0,44	-0,32
10	-0,34	-0,08	-0,19	-0,41	-0,31
CDK2					
Rank/Cosolvent	Acetamide	Benzene	Acetanilide	Imidazole	Isopropanol
1	-0,74	-0,94	-0,89	-0,92	-1,21
2	-0,67	-0,78	-0,87	-0,78	-1,01
3	-0,61	-0,68	-0,71	-0,75	-0,74
4	-0,57	-0,52	-0,63	-0,62	-62
5	-0,56	-0,46	-0,59	-0,58	-0,62
6	-0,55	-0,43	-0,57	-0,55	-0,53
7	-0,5	-0,41	-0,56	-0,55	-0,47
8	-0,5	-0,35	-0,54	-0,51	-0,46
9	-0,48	-0,35	-0,53	-0,51	-0,45

10	-0,45	-0,32	-0,52	-0,5	-0,44

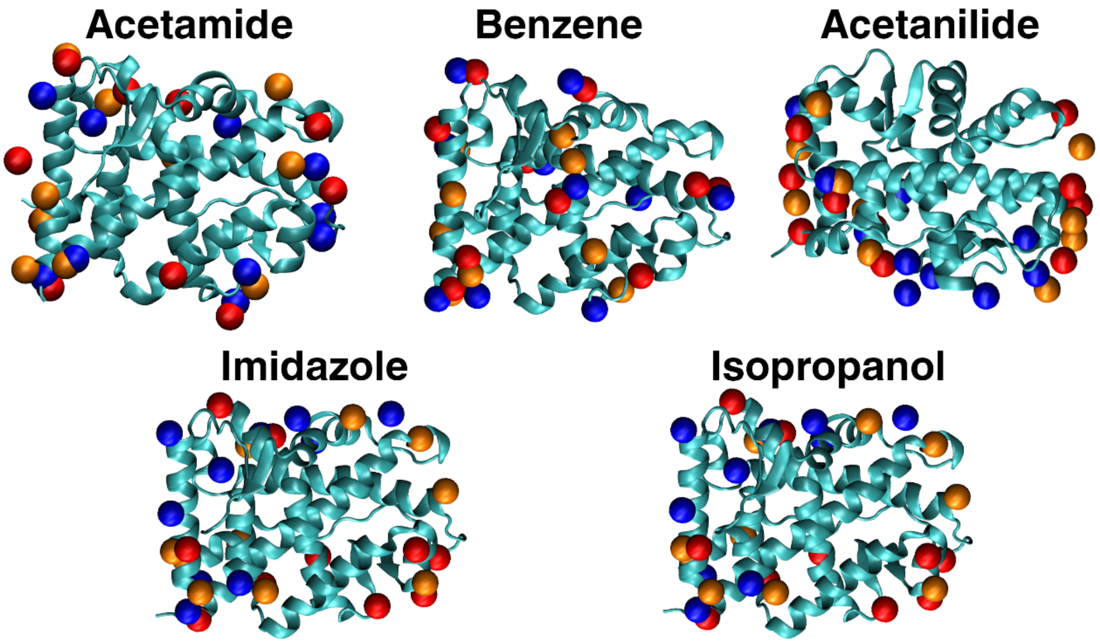

Supporting Figure 13: CAT clusters obtained for different replicas (depicted by red, orange, and blue spheres) for the androgen receptor ligand binding domain (AR-LBD) simulations in five different cosolvents. The cosolvents molecules in different replicas occupy similar regions on the protein surface.

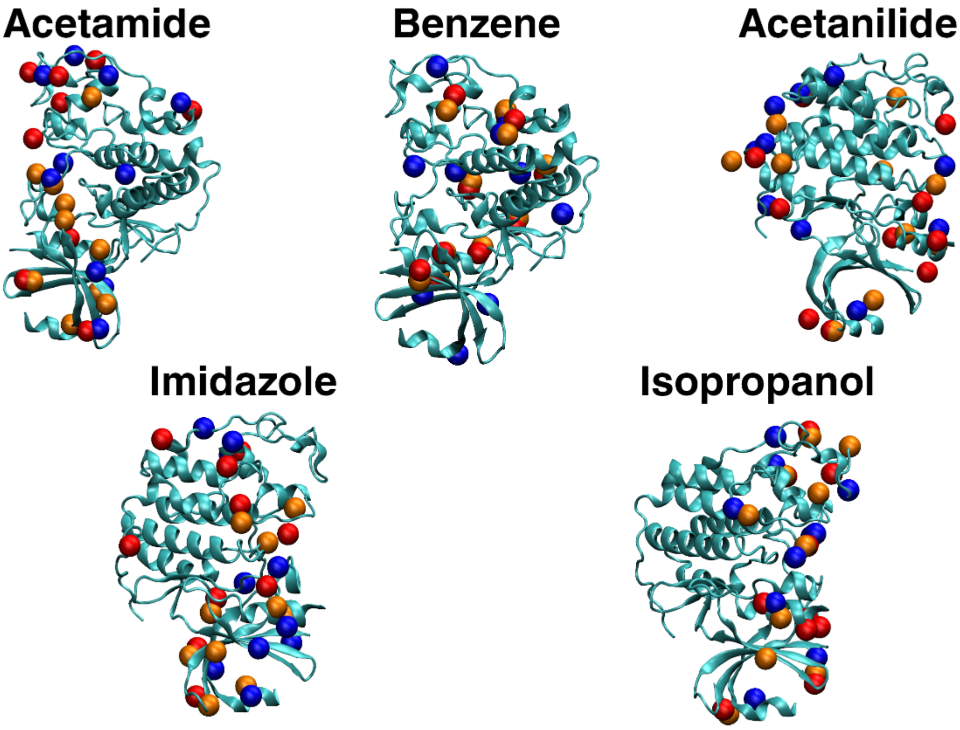

Supporting Figure 14: CAT clusters obtained for different replicas (depicted by red, orange, and blue spheres) for the CDK2 simulations in five different cosolvents. The cosolvents molecules in different replicas occupy similar regions on the protein surface.

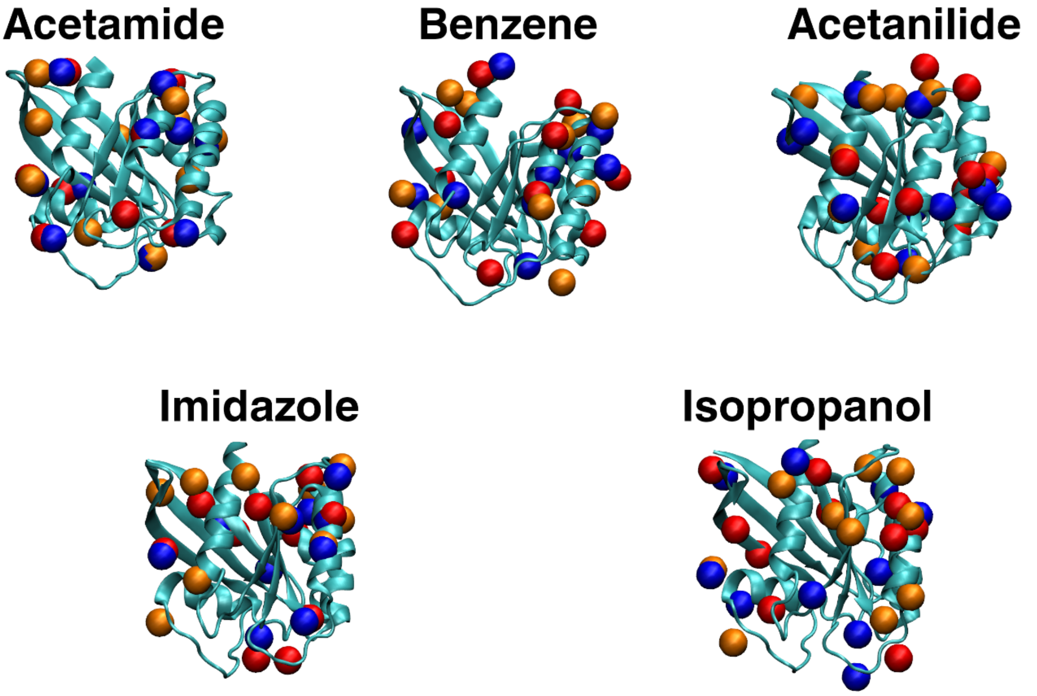

Supporting Figure 15: CAT clusters obtained for different replicas (depicted by red, orange, and blue spheres) for the HRas simulations in five different cosolvents. The cosolvents molecules in different replicas occupy similar regions on the protein surface.

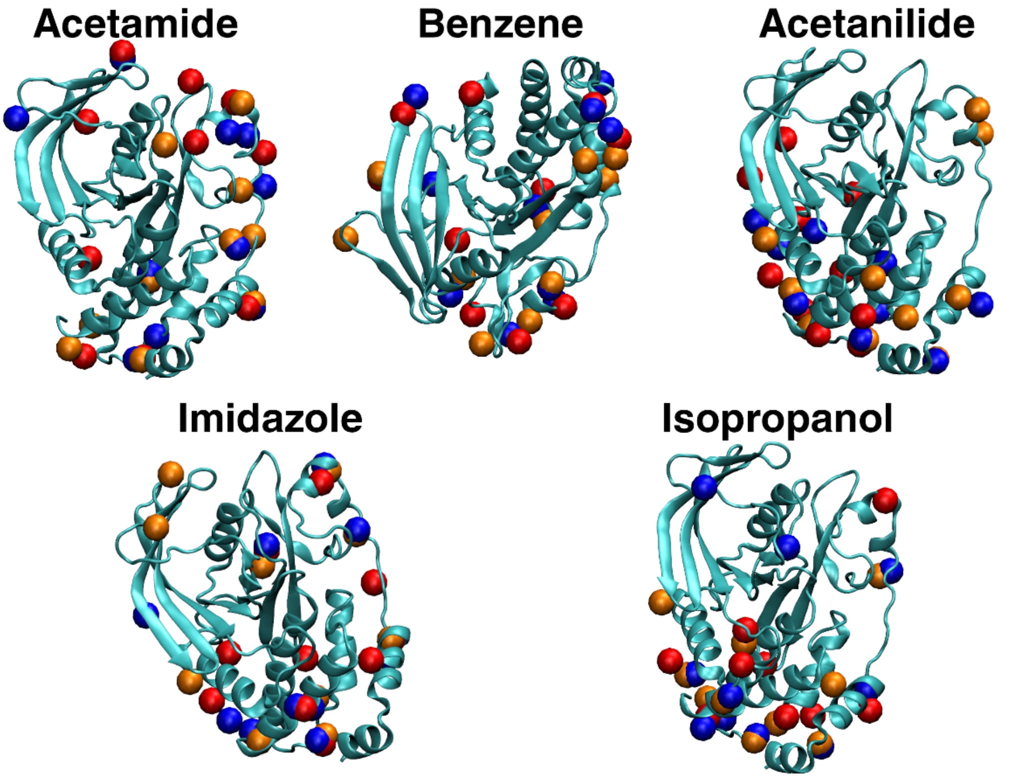

Supporting Figure 16: CAT clusters obtained for different replicas (depicted by red, orange, and blue spheres) for the PTP1B simulations in five different cosolvents. The cosolvents molecules in different replicas occupy similar regions on the protein surface.

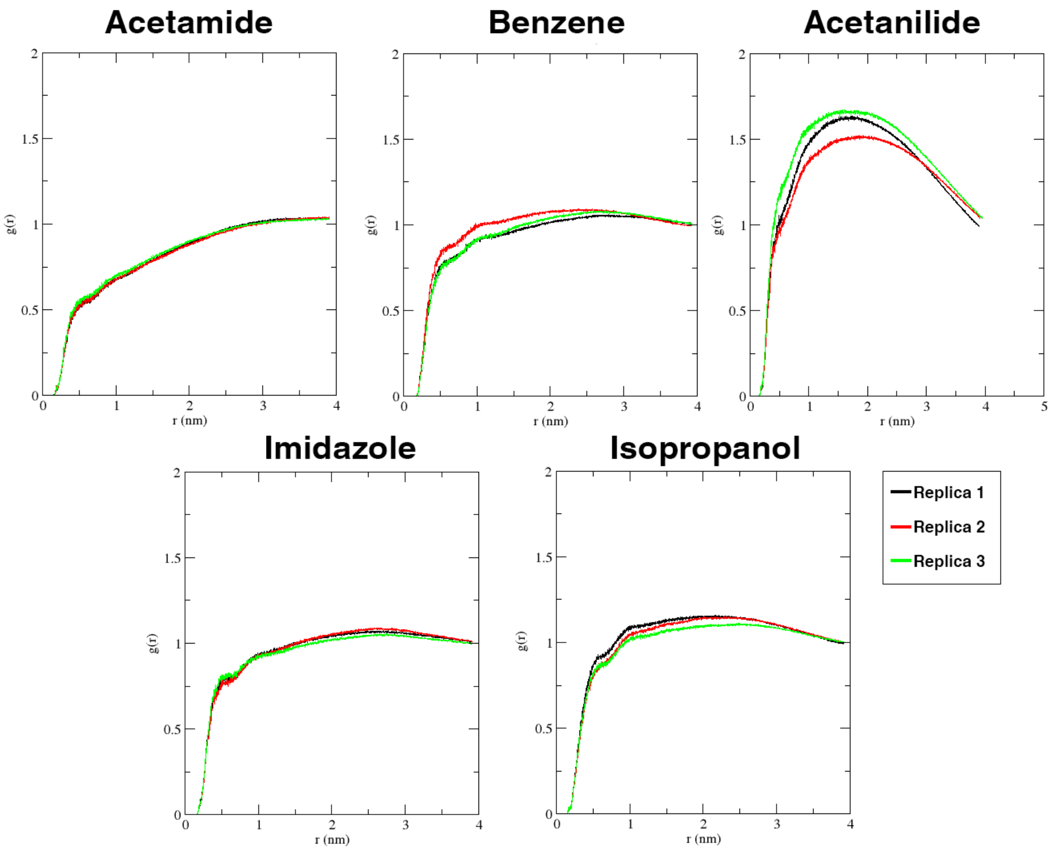

Supporting Figure 17: Protein-cosolvent radial distribution functions (RDF) calculated for different replicas of the ligand binding domain of human Androgen Receptor (AR-LBD) in five different cosolvents. Most of the cosolvent molecules in different replicas (black, red, and green) show similar RDFs. This indicates that different replicas sampled similar cosolvent shells surrounding the protein.

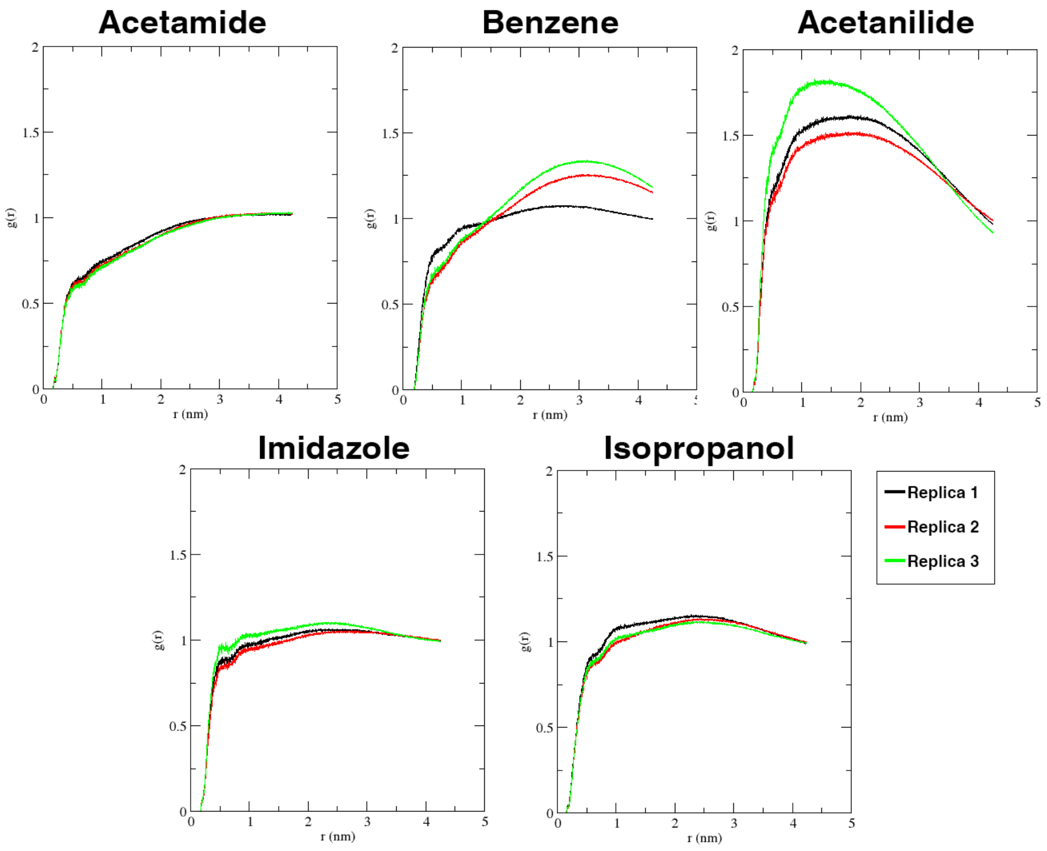

Supporting Figure 18: Protein-cosolvent radial distribution functions (RDF) calculated for different replicas of the CDK2 kinase in five different cosolvents. Most of the cosolvent molecules in different replicas (black, red, and green) show similar RDFs. This indicates that different replicas sampled similar cosolvent shells surrounding the protein.

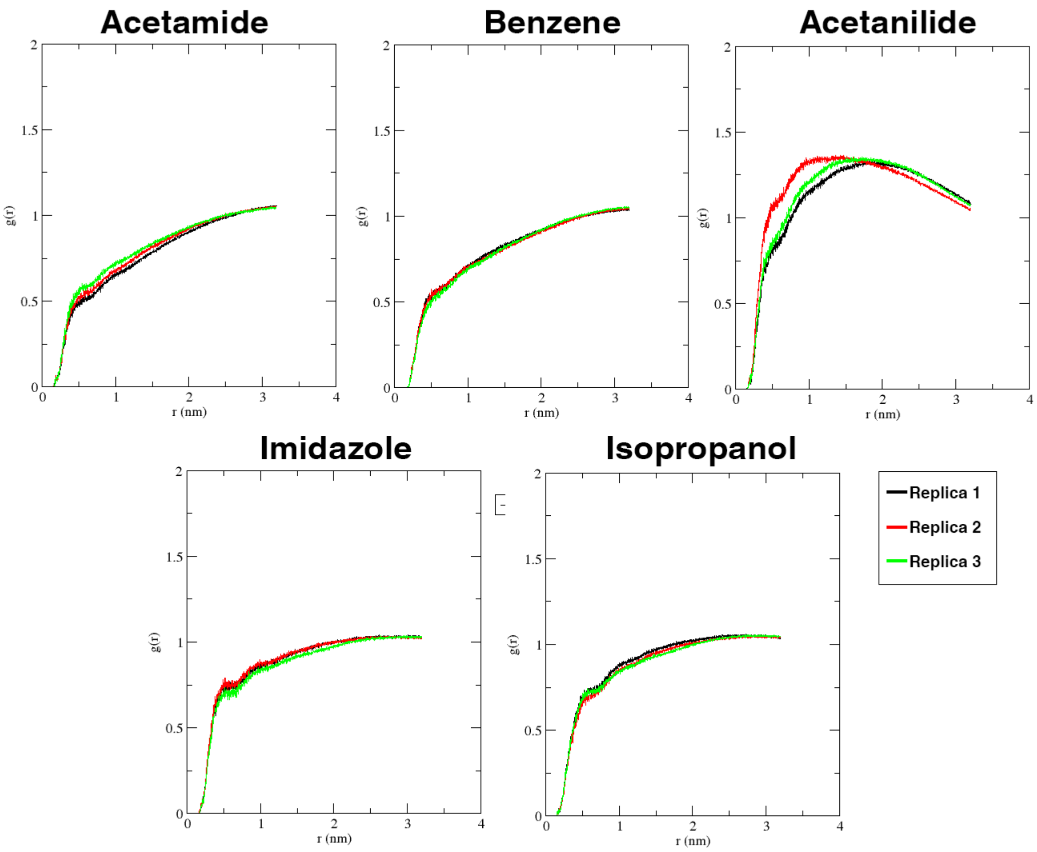

Supporting Figure 19: Protein-cosolvent radial distribution functions (RDF) calculated for different replicas of the HRas in five different cosolvents. Most of the cosolvent molecules in different replicas (black, red, and green) show similar RDFs. This indicates that different replicas sampled similar cosolvent shells surrounding the protein.

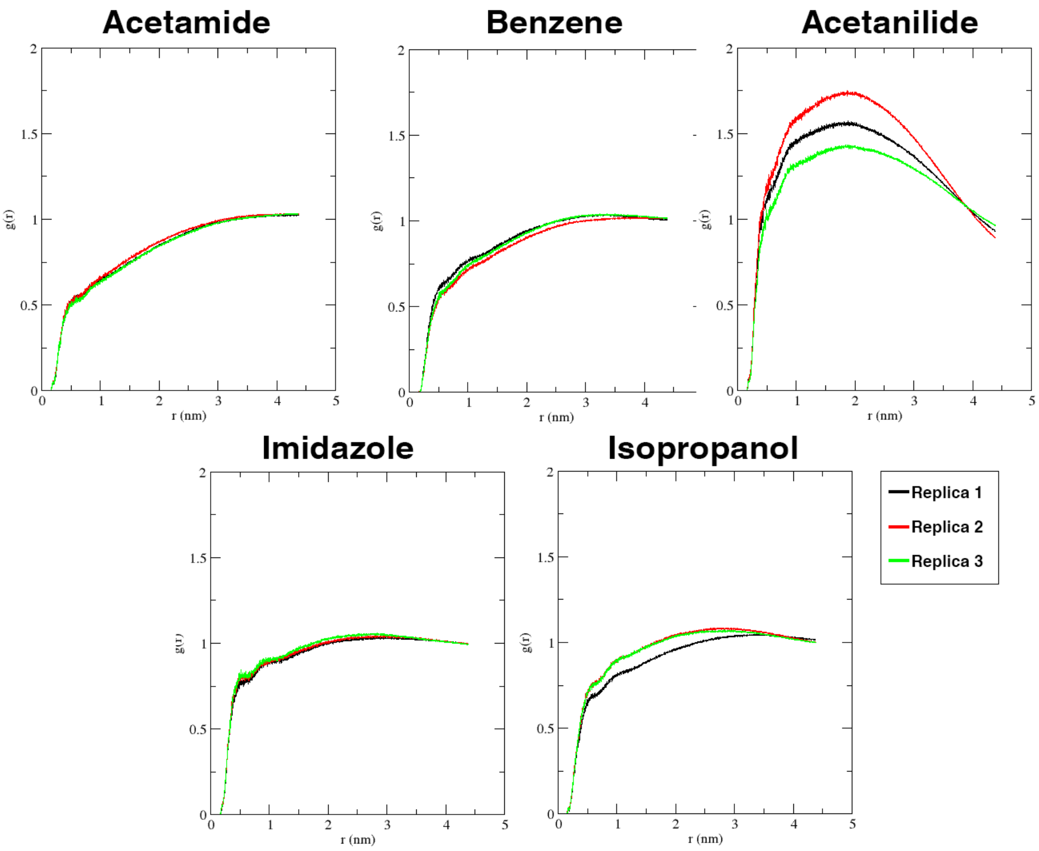

Supporting Figure 20: Protein-cosolvent radial distribution functions (RDF) calculated for different replicas of the PTP1B in five different cosolvents. Most of the cosolvent molecules in different replicas (black, red, and green) show similar RDFs. This indicates that different replicas sampled similar cosolvent shells surrounding the protein.
